# Health and non-health benefits and equity impacts of individual-level economic relief programs during epidemics/pandemics in high income settings: a scoping review

**DOI:** 10.1186/s12889-024-19493-8

**Published:** 2024-08-05

**Authors:** Adeteju Ogunbameru, Gebremedhin Beedemariam Gebretekle, Adrianna Perryman, Marian Hassan, Ashley Farrell, Kuan Liu, Sharmistha Mishra, Beate Sander

**Affiliations:** 1 Institute of Health Policy, Management and Evaluation, University of Toronto, Toronto, ON Canada; 2 Toronto Health Economics and Technology Assessment (THETA) Collaborative, University Health Network, Toronto, ON Canada; 3 School of Global Health, York University – Keele Campus, Toronto, ON Canada; 4 Library & Information Services, University Health Network, Toronto, ON Canada; 5 Dalla Lana School of Public Health, University of Toronto, Toronto, ON Canada; 6 Department of Medicine, Division of Infectious Disease, University of Toronto, Toronto, ON Canada; 7 Centre of Urban Health Solutions, St, Michael’s Hospital, Toronto, ON Canada; 8 Public Health Ontario, Toronto, ON Canada; 9 ICES, Toronto, ON Canada

**Keywords:** Pandemic preparedness, Epidemics, COVID-19, Infectious disease, Equity

## Abstract

**Background:**

Economic relief programs are strategies designed to sustain societal welfare and population health during a regional or global scale infectious disease outbreak. While economic relief programmes are considered essential during a regional or global health crisis, there is no clear consensus in the literature about their health and non-health benefits and their impact on promoting equity.

**Methods:**

We conducted a scoping review, searching eight electronic databases from January 01, 2001, to April 3, 2023, using text words and subject headings for recent pathogens (coronavirus (COVID-19), Ebola, Influenza, Middle East Respiratory Syndrome (MERS), severe acute respiratory syndrome (SARS), HIV, West Nile, and Zika), and economic relief programs; but restricted eligibility to high-income countries and selected diseases due to volume. Title and abstract screening were conducted by trained reviewers and Distiller AI software. Data were extracted in duplicates by two trained reviewers using a pretested form, and key findings were charted using a narrative approach.

**Results:**

We identified 27,263 de-duplicated records, of which 50 were eligible. Included studies were on COVID-19 and Influenza, published between 2014 and 2023. Zero eligible studies were on MERS, SARS, Zika, Ebola, or West Nile Virus. We identified seven program types of which cash transfer (*n* = 12) and vaccination or testing incentive (*n* = 9) were most common. Individual-level economic relief programs were reported to have varying degrees of impact on public health measures, and sometimes affected population health outcomes. Expanding paid sick leave programs had the highest number of studies reporting health-related outcomes and positively impacted public health measures (isolation, vaccination uptake) and health outcomes (case counts and the utilization of healthcare services). Equity impact was most often reported for cash transfer programs and incentive for vaccination programs. Positive effects on general well-being and non-health outcomes included improved mental well-being and quality of life, food security, financial resilience, and job security.

**Conclusions:**

Our findings suggest that individual-level economic relief programs can have significant impacts on public health measures, population health outcomes and equity. As countries prepare for future pandemics, our findings provide evidence to stakeholders to recognize health equity as a fundamental public health goal when designing pandemic preparedness policies.

**Supplementary Information:**

The online version contains supplementary material available at 10.1186/s12889-024-19493-8.

## Background

Infectious disease epidemics and pandemics can result in catastrophic economic collapse and disastrous human, social, and health consequences [1]. Populations experiencing social and economic marginalization have consistently experienced the highest risk of infection, disease severity and death [2–4]. Across high-income settings, racially minoritized individuals experienced disproportionate burden of COVID-19, H1N1 influenza, and tuberculosis [3, 5, 6]; driven by how systemic racism has shaped occupational risks, housing, and health care [6].

Individual-level economic relief programs are economic interventions implemented by governments, institutions, or private sources during an epidemic or pandemic to limit the disproportional health and economic consequences often experienced by populations at higher risk of the disease (e.g., low- and modest-income families, families with children, homeless population, and indigenous persons), support public measures and improve population health [7]. Programs implemented during the COVID-19 pandemic included paid sick leave, caregiver and childcare benefits, unemployment compensations for furloughed workers, and food supply and direct cash payments to low-income earners [7].

Pandemic-informed individual level economic relief policies are intended to encourage changes in human behaviour and motivate individuals to make healthy choices that impact health and overall well-being [8–10]. In a 2020 survey, 94% of respondents indicated compliance to a proposed two-week self -quarantine during the COVID-19 outbreak if financial compensation for lost wages is guaranteed; however, when the financial compensation option was removed, the compliance rate dropped to 57% [9]. Nevertheless, we found very limited evidence on the health-related benefits of individual level economic relief programs in our exploratory review, conducted in few electronic databases in September 2020 to assess the feasibility of a broader review.

Because the implementation individual level economic relief programs are often costly [7] and there is often a debate about their impact during outbreaks [11], we systematically chart their health and non-health benefits, and equity impacts to inform pandemic preparedness planning.

Our objective is to map the current state of the literature on individual-level economic relief programs during infectious disease outbreaks and their impact on the effectiveness of public health measures, individual and population health, non-health outcomes, and health equity during regional or global scale infectious disease outbreaks. Our review questions are:

1. What are the types of individual-level economic relief programs implemented during an infectious disease outbreak?
2. How and to what extent do pandemic/epidemic individual-level economic relief programs impact the effectiveness of public health measures during epidemics?
3. How and to what extent do changes in public health measures associated with pandemic/epidemic individual-level economic relief programs impact health outcomes?
4. Do health benefits associated with pandemic/epidemic individual-level economic relief programs differ across demographic and social groups, and place of residence? If so, how?
5. What are the non-health outcomes assessed in eligible studies identified?
6. What are the limitations associated with pandemic/epidemic individual-level economic relief programs?
7. What are the knowledge gaps in the literature in relation to the questions above?

## Methods

We followed the updated Arksey and O’Malley’s framework on conducting scoping review [12, 13] and the Preferred Reporting Items in Systematic Reviews and Meta-Analyses extension for Scoping Reviews (PRISMA-ScR) reporting guideline [14].

Our protocol is available at 10.1136/bmjopen-2021-057386 [15]. We made the following changes to the protocol: We excluded HIV publications, publications from low- and middle-income countries, and non-English publications due to their volume, constrained resources, and differences in health systems structure. Also, jurisdictional differences in the health benefits associated with pandemic/epidemic individual-level economic relief programmes were assessed by place of residence (e.g., urban or rural), not by country type.

### Search strategy

An information specialist developed our comprehensive search strategy using text words and Medical Subject Headings (MeSH) terms. In the original search strategy, from January 1, 2001, to October 8, 2021, we searched concepts relating to pandemic/epidemic infectious diseases (specifically, coronaviruses, influenza A, SARS, MERS, HIV/AIDS, Zika, Ebola and West Nile) and economic relief programs (e.g., government financing, public assistance, food assistance, medical assistance, workers compensation, social welfare, charities, and childcare). We restricted our search to post-2001 because of the global changes observed in living standards and health care delivery (now focused on primary health care) since countries implemented the Millennium Development Goals in 2001 [16]. We updated our search on April 3, 2023. For the updated search, we excluded HIV terms from the search strategy due to the volume of HIV/AIDS publications during the original screening. The original search strategy was developed in the Ovid Medline and then translated to other databases. Both MEDLINE search strategies are reported in the appendix.

We searched eight databases—MEDLINE, OVID E-pub Ahead of Print In-Process & Other Non-Indexed Citations, EMBASE, Cochrane CENTRAL, all on the OvidSP platform; EconLit, CINAHL, on the EBSCO platform; ISI Web of Science on the Clarivate platform, and Global Index Medicus from the World Health Organization (WHO). We manually searched the reference lists of eligible studies to ensure we do not miss relevant articles.

Our search excluded clinical conferences, comments, editorials, letters, and animal studies. No study design, language or country restriction was employed in our search strategy.

### Study screening and data extraction

De-duplication, and title and abstract screening were conducted using the Distiller SR software, including the artificial intelligence (AI) simulation tool that automates the title and abstract screening process [17]. Four trained reviewers conducted the title and abstract screening in parallel without using Distiller AI. Distiller AI was used at the end of the screening process to check for screening errors among excluded records. Retrieved records were tagged by country type (high-income or low-and-middle-income) and disease (HIV, COVID-19, Zika, Ebola, West Nile, MERS, SARS, or influenza A (H1N1 and H2N2). We excluded commentaries, book chapters, conference abstracts with no full text, study protocols, and business targeted economic relief programs.

**Fig. 1:**
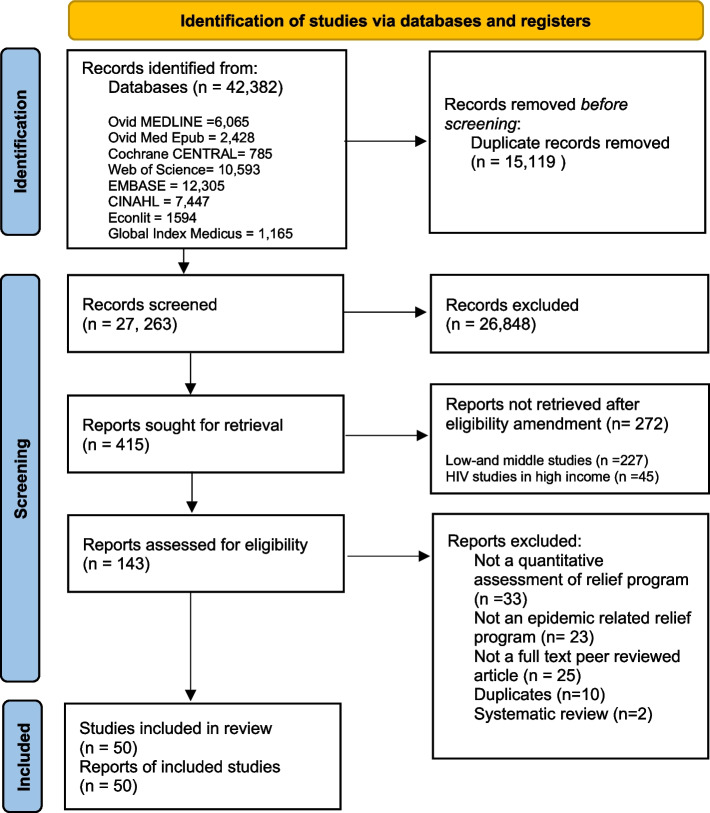
Preferred Reporting Items in Systematic Reviews and Meta-Analyses (PRISMA) flowchart

Following title and abstract screening, we restricted study eligibility to COVID-19, Zika, Ebola, West Nile, MERS, SARS, or influenza A (H1N1 and H2N2) and high-income settings due to volume (see Fig. 1 for details on the excluded studies). We also excluded non-English articles due to limited translation resources.

Full text screening and data extraction were conducted in duplicates by two trained reviewers using Microsoft Excel software. Disagreements were resolved through discussions until consensus was reached. Data abstraction was conducted using a pre-tested template. Data elements included study and population characteristics (study objective, study design, study population), infectious disease outbreak description (name of outbreak and time of outbreak), economic relief program description (eligible population, program and equity considerations used in assessing eligibility and program implementation time period), public health measure outcomes, population health outcomes, general health outcomes, equity impacts (by variables such as demographics, social and jurisdiction), and non-health outcomes.

Equity-impact of a disease and implemented interventions are often measured among groups or settings that are likely to be disadvantaged by the outbreak [5]. Factors that may be considered when measuring inequities include income, employment, and gender [5]. In our review, data extraction and synthesis on equity impact was guided by the PROGRESS-Plus framework to ensure that we systematically consider health equity under relevant dimensions, including demography, social factors, disability, and sexual orientation [18].

Individual level economic relief programs were categorized by the nature of the program and intended population. For examples, monetary incentive programs directed to increase vaccine uptake or testing were categorized as “incentive for vaccination or testing” program; fiscal stimulus directed to workers when ill/exposed to encourage quarantine/isolation was termed “expanded paid sick leave”; employment insurance payment to furloughed workers or unemployed individuals was termed “unemployed assistance”; direct cash payment to support individuals and households was termed “cash transfer” food supply programs to communities, schools, households was categorized as “food assistance”; two or more individual-level economic relief program groups simultaneously was termed “mixed program” and *“*others” program category which included subsidy program and expanded child tax credit program.

Full details of the data elements extracted is described in our protocol [13].

### Data charting

We summarized public health measures, health, non-health measures and equity impact associated with individual-level economic programs using a narrative approach and visual plots. In the equity-impact analysis, we provided details on how outcomes differed by equity variables.

## Results

Our initial and updated search yielded 27,263 de-duplicated records from the eight databases. After title and abstract screening, we retrieved 415 records for full-text screening. Following the amended eligibility criteria, we excluded 227 studies from low- and middle-income countries and 45 studies on HIV from high-income countries. We assessed the full text of 143 studies and excluded 93 records. The reasons for exclusions included: no quantitative assessment of the individual-level economic relief program (*n* = 33), not an individual-level economic relief program (*n* = 23), and book/commentary/report/opinion/ research letters/conference abstract/media release (*n* = 25). Fifty studies were found eligible for final review. The PRISMA flow diagram is shown in Fig. 1.

### Studies and program characteristics

Eligible studies were published between 2014 and 2023. Ninety percent (90%) were COVID-19 related (*n* = 45) and others were on influenza (*n* = 5). We did not identify eligible studies on MERS, SARS, Zika, Ebola, or West Nile virus. Seventy-three percent of included studies were conducted in the United States of America (*n* = 36) [19–53], followed by Japan [54–56], South Korea [46, 57, 58], Australia [59, 60], Canada [61, 62], Chile, Israel, Spain, and Singapore [63–66]. Two-third of eligible studies used a survey study design [20, 22, 25, 31, 32, 35–39, 46–50, 53–57, 43, 60, 62, 64, 66–69]; others included quasi-experiment [28, 44], simulation [23, 25, 30, 58, 63, 70], observational [29, 41, 52, 59], and randomized control trial design [45, 51, 61, 65].

We identified 28 unique individual-level economic relief programs among eligible studies and categorized them into seven groups.

**Fig. 2:**
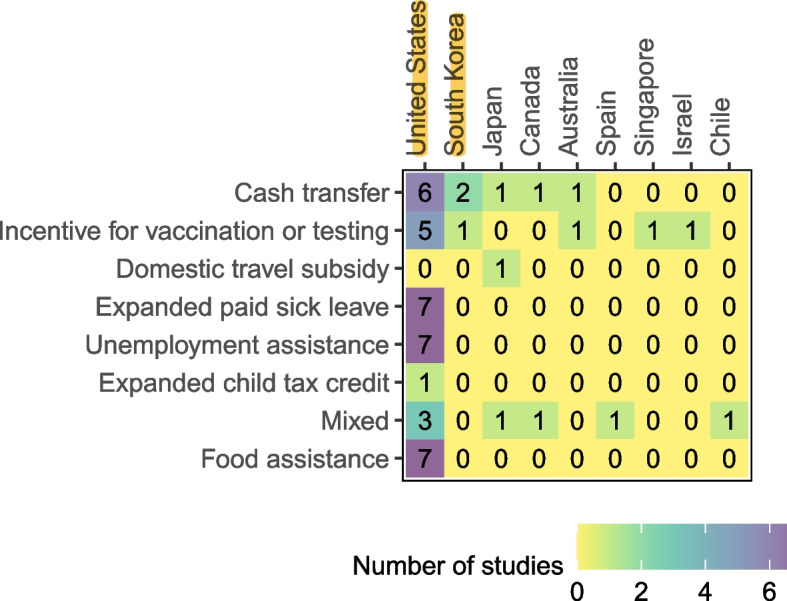
Heat map depicting program and studies characteristics of included studies. Footnotes: [The number of studies by individual-level economic relief program type and country are ordered from highest to lowest (left to right). We assigned dark to light colours to depict a no study scenario to having more than 6 studies scenarios]

Of the 50 eligible studies, three sets of seven studies reported on COVID-19 related unemployment assistance programs [21, 22, 25, 26, 29, 33, 50], COVID-19 food assistance programs [25, 27, 31, 43, 46, 47, 49], and expanded paid sick leave [22, 25, 37–39, 42, 52] in the context of COVID-19 pandemic and Influenza epidemic. Eleven eligible studies reported on COVID-19 cash transfer programs [20, 23, 48, 51, 53, 55, 57, 59, 61, 70, 71]; nine studies evaluated vaccine and testing incentives during COVID-19 pandemic and Influenza epidemic [32, 36, 40, 41, 45, 58, 60, 65, 66]; seven assessed mixed programs [30, 34, 35, 54, 62–64], three of which combined unemployment assistance and cash transfer programs [30, 34, 62]. Two COVID-19 studies reported on programs in the “other “category [44, 56]. Figure 2 presents the program and study characteristics as a heat map.

### Health, non-health, and equity outcomes of individual-level economic relief programs

**Fig. 3:**
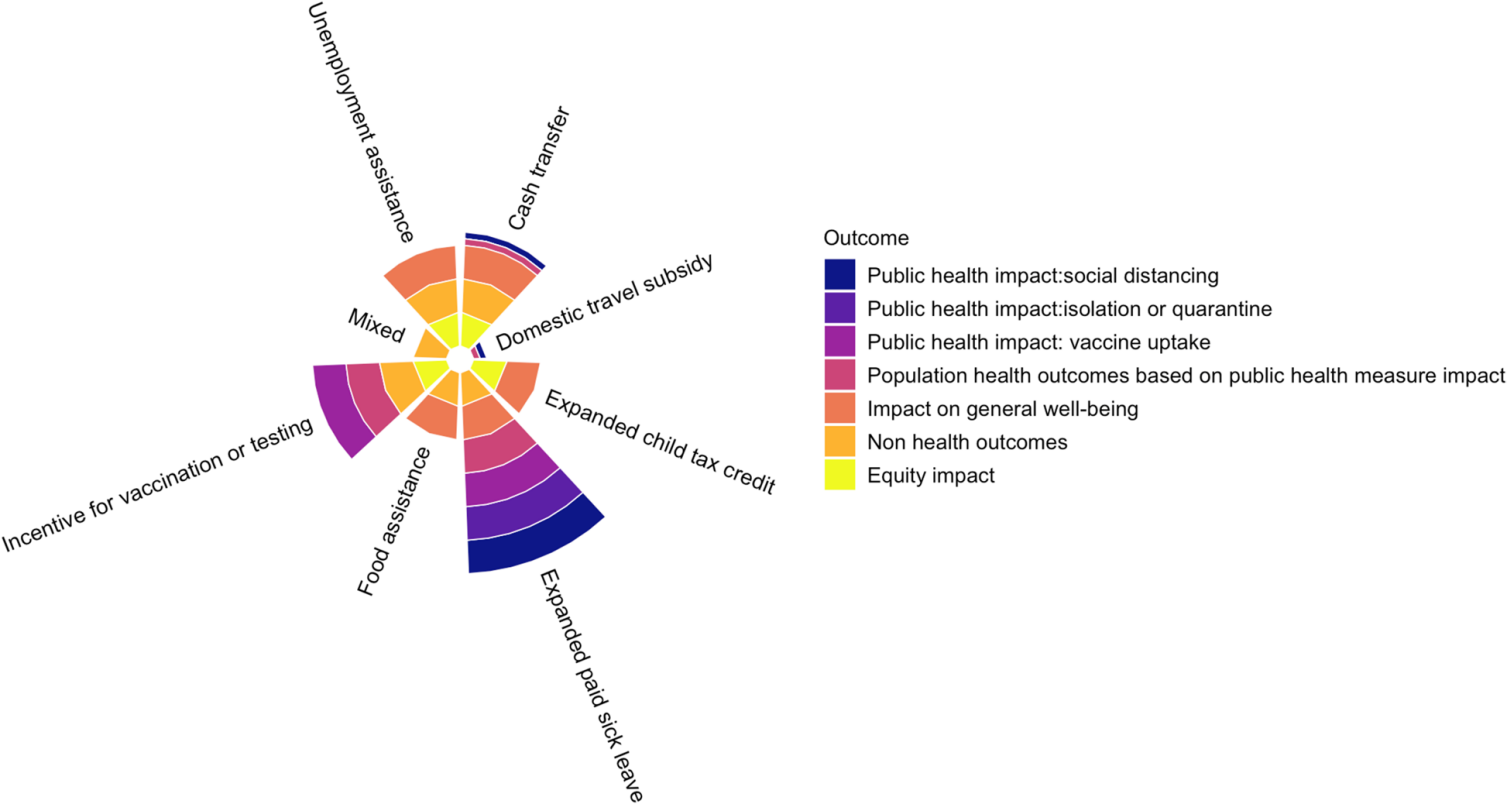
Coxcomb Chart depicting study outcomes by individual-level economic relief program type Footnotes: [The different colours of the ray indicate the various components of the review outcomes. The width of the ray indicates the impact of the individual-level economic relief program type on the components of the review outcomes. A wide width implies that the individual-level economic relief program type has a positive impact on the specific review outcome assessed and a narrow width denotes a negative impact.]

All eligible studies reported on at least one review outcome. Studies on expanding paid sick leave programs reported the most health-related outcomes (*n* = 7) [19, 28, 37–39, 42, 52]. The impact on non-health outcomes was commonly assessed for cash transfer programs (*n* = 10) [20, 23, 51, 53, 55, 57, 59, 61, 70, 71]. Incentive for vaccination program studies contributed majorly to the equity data (*n* = 3) [32, 45, 65]. Figure 3 presents a Coxcomb chart of study outcomes by individual-level economic relief program type.

#### Health impacts

Thirty-six of the 50 eligible studies were designed to evaluate the health impact of an economic relief program [19–21, 28, 32, 35–45, 48, 52, 54, 56–58, 60, 61, 65, 66].

*Impact on public health measures.* Fifteen out of 50 studies were designed to examine the effect of economic relief programs on public health measures [28, 32, 36, 37, 39–42, 45, 56, 60, 61, 65, 66, 71].

Three studies, reporting on programs of expanded paid sick leave (*n* = 1), domestic travel subsidy (*n* = 1), and cash transfer (*n* = 1), found that economic relief programs could have a positive, neutral, or negative impact on social distancing, depending on program type [42, 56, 61]. In two studies, expanded paid sick leave was associated with an increased probability of workers isolating when sick by 15% [28] and an increased amount of time spent away from work when sick by 1.10 days [38]. Expanding paid sick leave was linked to an increase in vaccination rates during influenza disease outbreaks by 10–15% [37, 39]. Vaccine incentives were reported to significantly increase vaccination rates by 7%—23.2% [32, 40, 65] in the context of COVID-19 and influenza outbreaks. A domestic travel subsidy program had no impact on masking practice, respiratory hygiene practice & surface disinfection [56].

*Population health outcomes based on programs’ impact on public health measures* were evaluated by eight studies [32, 39, 41, 42, 56, 58, 61]. Health outcomes reported on included symptom and case counts, infection transmission, healthcare visits and herd immunity. Herd immunity was measured by a multinomial model regressing the effect COVID-19 vaccine incentive on US population vaccination level. [32] The economic relief programs were cash transfers [61], expanded paid sick leave [19, 38, 39, 42], incentives for vaccination/testing [32, 58], and domestic travel subsidy [56]. Expanded paid sick leave and incentive for vaccination/testing were associated with positive health outcomes [19, 32, 38, 39, 42, 58] while cash transfer and domestic travel subsidy were linked to neutral [61] and negative health outcomes [56] respectively.

*General well-being* outcomes were reported in thirteen studies [20, 21, 35, 37, 39, 43, 44, 46, 48, 52, 54, 61, 66]. The economic relief program types assessed included cash transfer (*n* = 4), unemployment assistance (*n* = 1), paid sick leave (*n* = 1), food assistance program (*n* = 2) and expanded child tax credit (*n* = 1). Cash transfer, unemployment assistance, food assistance, and expanded child tax credit were reported to have a positive impact on mental well-being in seven studies [20, 21, 35, 44, 46, 48, 43]. Paid sick leave was associated with intention to seek medical care; food assistance programs were associated with healthier eating and improved intention to seek medical care when ill [37, 43].

#### Non-health impacts

Thirty-three of the 50 (60%) eligible studies reported non-health outcomes [20–23, 25, 26, 29, 31, 34, 39, 44, 55, 57, 61–64, 68–70, 72]. Economic relief programs had a positive impact on non-health measures including food security, financial resilience/savings/financial security, consumption spending and debt payment, housing/mortgage payment and job security. Programs included cash transfer (*n* = 10), unemployment assistance (*n* = 10), incentive for vaccination (*n* = 1), food assistance (*n* = 6), mixed (*n* = 4) and expanded paid sick leave (*n* = 2).

#### Equity impacts

Nine of the 50 (18%) eligible studies reported on the equity impact of the individual level economic relief programs [32, 35, 39, 44, 45, 48, 56, 61, 65]. Program types assessed included cash transfer (*n* = 2), incentive for vaccination (*n* = 3), mixed (*n* = 1), expanded paid sick leave (*n* = 1), and “other” (*n* = 2). The equity factors considered were demographics (age, and race), socioeconomic status (employment), and jurisdiction.

Health outcomes associated with cash transfer programs differed across demographic factors (race and age) in two studies [48, 61]. Cash transfer was association with reduced incidence of COVID-19 symptoms among 50 years or older [48] and improved financial savings (a proxy for improved mental health) among Hispanics [61].

In three studies assessing the effect of incentive for vaccination, health outcomes significantly differed by race, age, and employment [32, 45, 65]. Vaccine incentive increased vaccination rates among Blacks and non-working elderly in COVID-19 pandemic and influenza epidemic [32, 65]. Vaccine incentive reduced vaccine uptake among 40 years and older in a COVID-19 study [45].

Expanded child tax credit program was linked to reduced anxiety symptoms among Blacks and Hispanics [44]. Domestic travel subsidy was associated with an increase in the incidence of COVID-19 symptoms among young participants [56]. Table 1 presents the health, equity impact and non-health outcomes reported in eligible studies.

**Table 1:** Summary of the health and non-health outcomes, and equity-impact reported in included studies

Author, year	Country	Study Design	Economic relief program type	Public health measure impact	Population health outcomes associated with public health measure impact	General well-being	Non-health outcomes	Equity impact
Persaud 2021 [61]	Canada	Randomized controlled trial	Cash transfer	No difference in the number of close contacts outside of the household between groups (Rate ratio 1.10 95% CI 0.83 to 1.46)	No difference in symptom count after 2 weeks (ratio of means 0.83; 95% CI 0.56 to 1.24, p = 0.34)	Self-reported health did not differ between groups after 2 weeks	Cash transfers did not reduce food insecurity (80%vs 71%)	Incidence of COVID-19 like symptoms reduced in those aged 50 years or older, but not in those < 50 years (*p* = 0.005)
Tsai 2020 [20]	USA	Survey	Cash transfer	NA	NA	Economic Impact Payment recipients were more likely to have tested positive or been untested for COVID-19 and less likely to screen positive for current major depression, generalized anxiety disorder, past 2-week suicidal ideation, COVID-19 era-related stress, and any illicit drug use compared to those who did not receive the payment	Economic Impact Payment was associated with fewer problems paying daily expenses	NA
Pichler 2020 [19]	USA	Survey (difference in difference analysis)	Expanded paid sick leave	NA	Relative to the mean number of new cases for the control group, the study model predicted a decrease of 417 new COVID-19 cases (56%) after the introduction of Families First Coronavirus Response Act	NA	NA	NA
Berkowitz 2020 [21]	USA	Survey (difference in difference analysis)	Unemployment assistance	NA	NA	Receiving unemployment insurance benefits was associated with lower risk for unmet health-related social needs, and depressive and anxiety symptoms	The risk of food insufficiency was lower for those who received unemployment insurance compared to those who did not. ( p < 0.0001)	NA
Lee 2021 [57]	South Korea	Simulation	Incentive for testing	NA	Study epidemiological model predicted an increase in the proportion of confirmed infected patients out of unidentified infected people in the susceptible-unidentified infected-confirmed stimulated population	NA	NA	NA
Schneider 2021 [28]	USA	Quasi-experimental	Expanded paid sick leave	15% reduction in the proportion of workers who reported working while sick	NA	NA	NA	NA
Ikeda 2021[54]	Japan	Survey	Mixed	NA	NA	the Special Cash Payment was associated with better health related quality of life (95% CI): 0.05 (0.03 to 0.08)	NA	NA
Robertson 2021 [32]	USA	Survey	Incentive for vaccination	Coupled incentives increase estimated vaccination rates by 7–8%. (*P* = 0.03)	Program could bring the US vaccination levels from 58% to more than 65% —substantially closer to the rates needed to reach herd immunity	NA	NA	Moderate incentive ($1500) increased vaccine uptake among Black respondents to 68 per cent (± 16%). Similar trend was found for Latino respondents Low income was associated with low vaccine uptake. The middle-income groups appeared most responsive to the vaccine incentive
Miyawaki 2020 [56]	Japan	Survey	Domestic travel subsidy	Program participants were more likely than non-participants to engage in risky behaviour (visiting restaurants, bars/nightclubs, at least once); no impact on masking practice, respiratory hygiene practice & surface disinfection	Program participants exhibited higher incidence of high fever (adjusted OR: 1.83; 95% CI 1.34 to 2.48), sore throat (aOR 2.09; 95% CI 1.37 to 3.19), cough (aOR 1.96; 95% CI 1.26 to 3.01), headache (aOR 1.24; 95% CI 1.08 to 1.44) and smell and taste disorder aOR 1.98; 95% CI 1.15 to 3.40) compared with non-participants	NA	NA	Higher incidence rates of COVID-19 like symptoms were more salient among young program participants compared to non-participants
Fan 2020 [35]	USA	Survey	Mixed			The unemployed working-age population receiving government assistance had a higher frequency of feeling nervous, lonely, and hopeless compared to those without assistance	NA	Unemployed working-age population with government assistance residing in urban areas showed significantly higher frequency feeling nervous, lonely, and hopeless compared to their non-urban counterparts
Acharya 2021 [40]	USA	Survey	Incentive for vaccination	lottery programs were associated with an increase of an average 23.12% increment in the new daily vaccination rate	NA	NA	NA	NA
Algara 2023 [41]	USA	Retrospective observational	Incentive for vaccination	positively influence vaccine preferences among the mass public and all partisan groups	NA	NA	NA	NA
Andersen 2023 [42]	USA	Survey	Expanded paid sick leave	reduced the number of hours people were not at home by a 0.38 h or 22.9-min and the proportion of individuals away from home for more than eight hours per day declined by 1.8% point	Weekly COVID-19 incidence decreased by 7.7 log points	NA	NA	NA
Barr 2021 [43]	USA	Survey	Food assistance	NA	NA	The community-based free meal distribution program led to healthier eating and reduced stress among participants	NA	NA
Batra 2023 [44]	USA	Quasi-experimental	Expanded child tax credit	NA	NA	Program resulted in fewer depressive and anxiety symptoms among low-income adults. (13.3 percent reduction from baseline anxiety levels (25.5 percent)	NA	Adults of Black, Hispanic, and other racial and ethnic backgrounds demonstrated greater reductions in anxiety symptoms compared to non-Hispanic White adults with children
Jacobson 2022 [45]	USA	Randomized controlled trial	Incentive for vaccination	Increases vaccination rates between 1.0% and 1.6% points	NA	NA	NA	Financial incentives reduced vaccination rates in both older individuals (ages 40 and over) and those who indicated they supported Trump in the 2020 presidential election
Jun'd 2022[60]	Australia	Survey	Incentive for vaccination	Program participants were 2.27 (95% CI 1.73 to 2.99) times more likely to be vaccinated	NA	NA	NA	NA
Kim 2021 [46]	USA	Survey	Food assistance	NA	NA	Implementing SNAP subsidy after unemployment insurance expiration was predicted to lead to a threefold higher risk of anxiety and depressive symptoms among those experiencing considerable financial hardship versus no hardship (*P* < .001),	NA	NA
Liu 2023 [48]	USA	Survey	Cash transfer	NA	NA	There was a negative association between the amount of stimulus received and financial hardship (a proxy for mental well-being) experienced by respondents during the COVID-19 pandemic	NA	African American households were less likely to increase spending, Hispanic households were more likely to increase savings
Pollack 2023 [52]	USA	Longitudinal observational	Expanded paid sick leave	States with pre-existing paid sick leave policies exhibited a greater drop in mobility (*P* < 001)	NA	NA	NA	NA
Shmueli 2022 [66]	Israel	Survey	Incentive for vaccination	Program did not increase the probability of getting vaccination immediately	NA	Incentives such as monetary rewards or the green pass did not increase the probability of getting vaccination immediately	NA	NA
Dudley 2021 [36]	USA	Survey	Incentive for vaccination	the odds of reporting receiving influenza vaccine compared to the control group was 0.22 (95%CI: 0.09– 0.51)	NA	NA	NA	NA
Yue 2020 [65]	Singapore	Randomized controlled trial	Incentive for vaccination	increased participation in vaccination from 4.5% to 7.5% (*P* = 0.001)	NA	NA	NA	The effect of increasing incentives on influenza vaccination rates was significant in nonworking elderly group than those who worked(*P* = < 0.001) and differed by ethnicity (*P* = 0.001), socio-economic status(*P* = < 0.0001), household size (0.009), and a measure of social resilience (*P* < 0.001)
Zhai 2018 [37]	USA	Survey	Expanded paid sick leave	Compared to workers with no paid sick leave, influenza vaccination coverage estimates for workers with paid sick leave was approximately 10–15% higher	NA	Paid sick leave was independently associated with seeking treatment for influenza illness (adjusted prevalence ratio (95%CI) 1.21 (1.01, 1.44)	NA	NA
Asfaw 2017 [38]	USA	Survey	Expanded paid sick leave	workers with paid sick leave spent an average of 1.10 more days away from work per year due to illness or injury (95% CI: 0.90 to 1.30)]	Assuming 10% to 12% of days absent due to illness, 0.026% transmission rate, and an average daily contact of three to five coworkers, a worker at work due to lack of paid sick leave could infect on the average 0.0405 to 0.0810 coworkers per year	NA	Paid sick leave could save employers $0.63 to $1.88 billion in reduced illness- related absenteeism costs per year	NA
Wilson 2014 [39]	USA	Survey	Expanded paid sick leave	Adjusted odds of having a vaccination increased with paid leave vs. without paid leave (OR = 1.42, CI: 1.31–1.53)	Decreased the number of influenza cases by 57 thousand	Universal paid leave is predicted to result in 18.2 thousand fewer healthcare visits, for the flu annually	Universal paid leave averted 63.8 thousand workdays lost to influenza each year,	White non-Hispanics had higher odds of receiving flu vaccination than another race/ethnicity
Marinescu 2021 [22]	USA	Survey	Unemployment assistance	NA	NA	NA	10% increase in unemployment benefits resulted in 3.6% decline in job applications	NA
Kim 2021 [57]	South Korea	Survey	Cash transfer	NA	NA	NA	Cash voucher scheme increased consumption spending among 36% of the households	NA
Bienvenido-Huertas 2021 [63]	Spain	Simulation	Mixed	NA	NA	NA	Unemployment aids could contribute to alleviating energy poverty, especially if the unemployed individual worked in a poorly paying job or for just a few hours	NA
Han 2020 [23]	USA	Simulation	Cash transfer	NA	NA	NA	Government cash program resulted in a decline in poverty during the COVID-19 pandemic	NA
Karger 2020 [70]	USA	Simulation	Cash transfer	NA	NA	NA	$1,200 stimulus payment disbursed increased consumer spending by $546	NA
Fan 2020 [25, 35]	USA	Simulation	Unemployment assistance	NA	NA	NA	The CARES Unemployment Insurance policies reduced cumulative deaths by 4.9%	NA
Evangelist 2022 [26]	USA	Survey	Unemployment assistance	NA	NA	NA	the unemployment insurance declined health care services spending declined by 1%	NA
Stiemele 2021 [68]	USA	Survey	Food assistance	NA	NA	NA	Food insecurity decreased most among those who received the local food assistance program	NA
Raifman 2021 [29]	USA	Retrospective observational	Unemployment assistance	NA	NA	NA	unemployment insurance was associated with a 4.3% decrease in food insecurity and a 5.7% decrease in eating less due to financial constraints	NA
Kubota 2021 [55]	Japan	Survey	Cash transfer	NA	NA	NA	Cash payments increased was associated with a jump in spending rate	NA
Fang 2020 [25]	USA	Survey	Food assistance	NA	NA	NA	The alternative school meals improved with food security for lower-income families	NA
Martin 2020 [30]	USA	Simulation	Mixed	NA	NA	NA	Social protection programs reduced the increase in poverty rates	NA
Clay 2021 [31]	USA	Survey	Food assistance	NA	NA	NA	Food assistance programs were associated with a higher likelihood of experiencing food insecurity (OR: 2.59 (1.62,4.16)	NA
Raifman 2020 [33]	USA	Survey	Unemployment assistance	NA	NA	NA	unemployment insurance was associated with a 4.4% decline in food insecurity, and 6.1% decline in eating less due to financial constraints	NA
Bhutta, 2020 [34]	USA	Survey	Mixed	NA	NA	NA	The CARES Act improved households’ financial security	NA
Men 2022 [62]	Canada	Survey	Mixed	NA	NA	NA	The applicants of Canada Economic Response Benefit, regular Employment Insurance benefits, and other Employment Insurance benefits had 2.53, 1.80, and 3.01 times higher adjusted odds of food insecurity, respectively, than non-applicants	NA
Madeira 2021 [64]	Chile	Survey	Mixed	NA	NA	NA	Income and expenses support program represented 13.6% of the average households’ permanent income of poor families in April 2020	NA
Breunig 2023 [59]	Australia	Retrospective observational	Cash transfer	NA	NA	NA	Program recipient had 46% of their weekly pre‐COVID‐19 wages replaced by the transfers	NA
Choi 2022 [71]	South Korea	Survey	Cash transfer	NA	NA	NA	The stimulus payments increased local consumption in establishments accepting the Gyeonggi local currency relative to other establishments	NA
Li 2022 [47]	USA	Survey	Food assistance	NA	NA	NA	SNAP significantly reduced food insecurity by 24.5% among households who were already food insufficient before the pandemic and by 11.9% for households with children	NA
Lowery 2022 [49]	USA	Survey	Food assistance	NA	NA	NA	Healthy Helping program was associated with a $26.95 increase in monthly spending on fruit, vegetables, nuts, and legumes and other shifts in the composition of food purchases	NA
Park 2023 [50]	USA	Survey	Unemployment assistance	NA	NA	NA	Unemployment insurance reduced mortgage debt payments	NA
Pilkauskas 2023 [51]	USA	Randomized controlled trial	Cash transfer	NA	NA	The cash transfer program had no impact on mental health	The cash transfer program had no impact on material hardship, parenting, child behavior, partner relationships, hardship avoidance, consumption, employment, and benefit use	NA
Wahdat 2022 [53]	USA	Survey	Cash transfer	NA	NA	NA	Economic Impact Payment was associated with 9.2% decrease in food insufficiency	NA

## Limitations associated with individual-level economic relief programs

Three studies discussed the limitations of their respective programs [21, 30, 57]. Limitations associated with unemployment assistance programs in two studies included state-level variability in unemployment insurance benefits [21], the complexity of the unemployment insurance program structure, the presence of barriers that prevent eligible individuals from receiving program benefits [21], and issues related to eligibility and implementation challenges, such as erroneous data on unemployment rate [30]. In a COVID-19 cash transfer program study, the authors suggested that the implemented consumption voucher program could constrain consumer choice and possibly harm consumer welfare and economic efficiency in the long run because beneficiaries could only redeem the vouchers at small business stores [57].

## Knowledge gap

Our review identified four key knowledge gaps: 

*Limited disease focus*. All eligible studies were on COVID-19 and influenza. Despite the occurrence of SARS, H1N1 influenza, MERS, Ebola, Zika and West Nile Virus outbreaks during eligibility period, we did not identify any eligible study on these outbreaks in our review.

*Lack of evidence on the effect of unemployment assistance programs and food assistance programs on public health measures* (such as physical distancing, quarantine/isolation, vaccination). Among the unemployment assistance programs and food assistance programs studies included in our review, none reported on public health measure impact.

*Limited evidence of the impact of individual-level economic relief programs on equity*Only 16% of eligible studies reported on the equity impact. None of the eight studies that assessed the equity impact of unemployment assistance programs, vaccine incentive programs, “other” programs, and cash transfer programs reported the program's effect on relevant equity variables. The impact of expanded paid sick leave programs and food assistance programs on equity remains undetermined.

*Lack of evidence on the long-term effect of individual-level economic relief programs on health outcomes,* which were not considered in the 36 eligible studies that reported health outcomes. Assessing the long-term health effects of individual-level economic relief policies, particularly post-pandemic during the recovery stage, could provide insight into the importance of the programs to equity and societal well-being.

## Discussion

Our review charts the current state of the literature on the types of individual-level economic relief programs implemented during infectious disease outbreaks.

Expanded paid sick leave was found to have a consistent pattern of improved health outcomes. Our findings corroborate the findings of a recent meta-analysis study of 12 studies, reporting that paid sick leave was associated with increased odds of following public health directives (vaccine uptake) and seeking medical care [73].

Individual-level economic relief programs improved population health outcomes among equity-deserving populations. The improved health outcomes observed among equity seeking populations is likely due to their improved adherence to public health measures enabled by economic relief programs. [74].

Furthermore, most individual-level economic relief program types positively impacted mental health, re-affirming the long-established evidence of the relationship between financial well-being and psychological well-being [72, 75]. Non-health outcomes associated with the programs had beneficial impact on key domains of social determinants of health i.e., economic stability and healthcare access and quality. Addressing social determinants of health is fundamental for improving health and reducing longstanding inequities in societal health [76].

Lastly, we identified four knowledge gaps which could help in priority setting of future research. Tailoring future research to address gaps would provide a more wholistic view to the robust and comprehensive impact of individual-level economic relief programs on health and equity during pre- and post-pandemic periods.

Our review has some limitations. First, we restricted our search strategy to studies in high-income countries due to the high volume of studies. Future reviews should synthesize the impact of similar programs in low-and middle-income countries. Second, due to the volume of studies identified we were only able to focus on selected infectious diseases. This limitation makes our findings disease-specific, requiring careful interpretation if attempting to extrapolate findings to other infectious diseases (e.g., HIV). Further, we did not search the grey literature and only included publications in English. Our findings may therefore be biased towards English-speaking high-income settings.

Our review’s strength lies in our use of a rigorous scoping review methodology. Screening and data extraction forms were pretested by all reviewers and revised as needed to ensure they are adequately sensitive to capture outcomes in eligible studies. We searched multiple relevant electronic scientific databases to ensure our results were comprehensive and accurate. Lastly, our eligibility criteria had no restriction on study design.

## Policy implications

Our findings provide compelling evidence that shows that individual-level economic relief programs are valuable, and their importance transcends health in pandemics. Although individual-level economic relief programs are capital-intensive, their broad and positive impact on public health measures, population health, general well-being, equity, and social determinants of health (e.g., economic stability) may make investing in them worthwhile.

As countries prepare for future pandemics, our findings provide evidence to stakeholders to recognize health equity as a fundamental public health goal when designing pandemic preparedness policies. Further, expansion of well-designed, robust, social safety net programs (such as individual level economic relief programs) for equity-deserving populations should be considered since evidence show that these programs not only improve health equity and social needs, but they may also address social determinants of heath.

## Conclusion

Individual-level economic relief programs implemented during epidemics/pandemics significantly impacted public health measures, outbreak-related population health outcomes and health equity. Our study findings can help inform investment decisions on individual-level economic relief programs to protect population health in future pandemics, particularly for equity-seeking populations, to prevent the widening of pre-existing societal inequity.

### Supplementary Information

Supplementary Material 1. Ovid Medline search strategy from January 1, 2001, to October 8, 2021.

Supplementary Material 2. Updated Ovid Medline search strategy from January 1, 2021, April 3, 2023.

Supplementary Material 3.  A comprehensive dataset of the outcomes extracted from the 50 eligible studies.

## Data Availability

All data generated or analysed during this study are included in this published article and its supplementary information files.
