# Supplementary material for: Health and non-health benefits and equity impacts of individual-level economic relief programs during epidemics/pandemics in high income settings: a scoping review
Source: BMC Public Health. 2024 Aug 5;24:2106. doi: 10.1186/s12889-024-19493-8 (PMC11299345; doi:10.1186/s12889-024-19493-8)
Supplement: Supplementary file 1 — Supplementary Material 1. Ovid Medline search strategy from January 1, 2001, to October 8, 2021. [file 12889_2024_19493_MOESM1_ESM.docx]

| **Ovid MEDLINE(R) <1946 to October 07, 2021>**   \| Search history sorted by search number ascending \| \| \| \| \| --- \| --- \| --- \| --- \| \| **#** \| **Searches** \| **Results** \| **Type** \| \|  \| \| \| \| \| \| \| \| 1 \| Epidemics/ \| 12206 \| Advanced \|  \|  \|  \| \| 2 \| Pandemics/ \| 67464 \| Advanced \|  \|  \|  \| \| 3 \| epidemic?.mp,kw,kf. \| 107187 \| Advanced \|  \|  \|  \| \| 4 \| pandemic?.mp,kw,kf. \| 101105 \| Advanced \|  \|  \|  \| \| 5 \| ((infectious or communicable) adj3 disease? adj3 outbreak*).mp,kw,kf. \| 1920 \| Advanced \|  \|  \|  \| \| 6 \| or/1-5 \| 200623 \| Advanced \|  \|  \|  \| \| 7 \| exp Coronavirus/ \| 98756 \| Advanced \|  \|  \|  \| \| 8 \| exp Coronavirus Infections/ \| 121075 \| Advanced \|  \|  \|  \| \| 9 \| Severe Acute Respiratory Syndrome/ \| 5615 \| Advanced \|  \|  \|  \| \| 10 \| SARS-CoV-2/ \| 86078 \| Advanced \|  \|  \|  \| \| 11 \| SARS Virus/ \| 3965 \| Advanced \|  \|  \|  \| \| 12 \| Middle East Respiratory Syndrome Coronavirus/ \| 1709 \| Advanced \|  \|  \|  \| \| 13 \| COVID-19/ \| 110403 \| Advanced \|  \|  \|  \| \| 14 \| (coronavirus* or corona virus*).mp,kw,kf. \| 80640 \| Advanced \|  \|  \|  \| \| 15 \| (coronavirinae* or corona virinae*).mp,kw,kf. \| 22 \| Advanced \|  \|  \|  \| \| 16 \| (txid1898672 or txid 1898672).mp,kw,kf. \| 0 \| Advanced \|  \|  \|  \| \| 17 \| alphacoronavirus*.mp,kw,kf. \| 241 \| Advanced \|  \|  \|  \| \| 18 \| (txid693996 or txid 693996).mp,kw,kf. \| 0 \| Advanced \|  \|  \|  \| \| 19 \| betacoronavirus*.mp,kw,kf. \| 33633 \| Advanced \|  \|  \|  \| \| 20 \| (txid694002 or txid 694002).mp,kw,kf. \| 0 \| Advanced \|  \|  \|  \| \| 21 \| covid*.mp,kw,kf. \| 113709 \| Advanced \|  \|  \|  \| \| 22 \| ncovid*.mp,kw,kf. \| 20 \| Advanced \|  \|  \|  \| \| 23 \| HCoV*.mp,kw,kf. \| 900 \| Advanced \|  \|  \|  \| \| 24 \| NCoV????.mp,kw,kf. \| 1521 \| Advanced \|  \|  \|  \| \| 25 \| (CoV2* or CoV-2*).mp,kw,kf. \| 92671 \| Advanced \|  \|  \|  \| \| 26 \| (CoV19 or CoV-19).mp,kw,kf. \| 90 \| Advanced \|  \|  \|  \| \| 27 \| (CoV2019 or CoV-2019).mp,kw,kf. \| 17 \| Advanced \|  \|  \|  \| \| 28 \| (2019nCoV or 2019-nCoV).mp,kw,kf. \| 1240 \| Advanced \|  \|  \|  \| \| 29 \| (SARS-CoV* or SARSCoV*).mp,kw,kf. \| 94999 \| Advanced \|  \|  \|  \| \| 30 \| (2019nCoV* or 2019-nCoV*).mp,kw,kf. \| 1253 \| Advanced \|  \|  \|  \| \| 31 \| (SARS or SARSr).mp,kw,kf. \| 101242 \| Advanced \|  \|  \|  \| \| 32 \| (severe acute respiratory adj2 syndrome*).mp,kw,kf. \| 21088 \| Advanced \|  \|  \|  \| \| 33 \| (txid694009 or txid 694009).mp,kw,kf. \| 0 \| Advanced \|  \|  \|  \| \| 34 \| (middle eastern respiratory adj2 syndrome*).mp,kw,kf. \| 64 \| Advanced \|  \|  \|  \| \| 35 \| MERS.mp,kw,kf. \| 5298 \| Advanced \|  \|  \|  \| \| 36 \| (MERSCoV* or MERS-Cov*).mp,kw,kf. \| 2373 \| Advanced \|  \|  \|  \| \| 37 \| (txid1335626 or txid 1335626).mp,kw,kf. \| 0 \| Advanced \|  \|  \|  \| \| 38 \| or/7-37 \| 138843 \| Advanced \|  \|  \|  \| \| 39 \| Influenza A virus/ \| 21658 \| Advanced \|  \|  \|  \| \| 40 \| Influenza A Virus, H1N1 Subtype/ \| 16349 \| Advanced \|  \|  \|  \| \| 41 \| Influenza A Virus, H2N2 Subtype/ \| 233 \| Advanced \|  \|  \|  \| \| 42 \| Influenza A Virus, H3N2 Subtype/ \| 4531 \| Advanced \|  \|  \|  \| \| 43 \| Influenza Pandemic, 1918-1919/ \| 205 \| Advanced \|  \|  \|  \| \| 44 \| influenza A.mp,kw,kf. \| 52360 \| Advanced \|  \|  \|  \| \| 45 \| flu A.mp,kw,kf. \| 407 \| Advanced \|  \|  \|  \| \| 46 \| fowl plague virus*.mp,kw,kf. \| 379 \| Advanced \|  \|  \|  \| \| 47 \| grippe.mp,kw,kf. \| 1273 \| Advanced \|  \|  \|  \| \| 48 \| pestis galli myxovirus*.mp,kw,kf. \| 0 \| Advanced \|  \|  \|  \| \| 49 \| orthomyxovirus*.mp,kw,kf. \| 334 \| Advanced \|  \|  \|  \| \| 50 \| (txid11320 or txid 11320).mp,kw,kf. \| 0 \| Advanced \|  \|  \|  \| \| 51 \| H1N1.mp,kw,kf. \| 20601 \| Advanced \|  \|  \|  \| \| 52 \| (spanish adj2 (influenza?? or flu)).mp,kw,kf. \| 467 \| Advanced \|  \|  \|  \| \| 53 \| (("1918" or "1919") adj2 (influenza?? or flu)).mp,kw,kf. \| 916 \| Advanced \|  \|  \|  \| \| 54 \| (russian* adj2 (influenza?? or flu)).mp,kw,kf. \| 36 \| Advanced \|  \|  \|  \| \| 55 \| ("1977" adj2 (influenza?? or flu)).mp,kw,kf. \| 35 \| Advanced \|  \|  \|  \| \| 56 \| (swine adj2 (influenza?? or flu)).mp,kw,kf. \| 2899 \| Advanced \|  \|  \|  \| \| 57 \| ("2009" adj2 (influenza?? or flu)).mp,kw,kf. \| 3461 \| Advanced \|  \|  \|  \| \| 58 \| (txid114727 or txid 114727).mp,kw,kf. \| 0 \| Advanced \|  \|  \|  \| \| 59 \| H2N2.mp,kw,kf. \| 667 \| Advanced \|  \|  \|  \| \| 60 \| (asian adj2 (influenza?? or flu)).mp,kw,kf. \| 598 \| Advanced \|  \|  \|  \| \| 61 \| (("1957" or "1958") adj2 (influenza?? or flu)).mp,kw,kf. \| 155 \| Advanced \|  \|  \|  \| \| 62 \| H3N2.mp,kw,kf. \| 7707 \| Advanced \|  \|  \|  \| \| 63 \| (hong kong adj2 (influenza?? or flu)).mp,kw,kf. \| 544 \| Advanced \|  \|  \|  \| \| 64 \| (("1968" or "1969") adj2 (influenza?? or flu)).mp,kw,kf. \| 107 \| Advanced \|  \|  \|  \| \| 65 \| (fujian adj2 (influenza?? or flu)).mp,kw,kf. \| 16 \| Advanced \|  \|  \|  \| \| 66 \| (("2003" or "2004") adj2 (influenza?? or flu)).mp,kw,kf. \| 382 \| Advanced \|  \|  \|  \| \| 67 \| (txid119210 or txid 119210).mp,kw,kf. \| 0 \| Advanced \|  \|  \|  \| \| 68 \| or/39-67 \| 57417 \| Advanced \|  \|  \|  \| \| 69 \| exp HIV Infections/ \| 296880 \| Advanced \|  \|  \|  \| \| 70 \| exp HIV/ \| 102998 \| Advanced \|  \|  \|  \| \| 71 \| HIV Long-Term Survivors/ \| 789 \| Advanced \|  \|  \|  \| \| 72 \| HIV Testing/ \| 336 \| Advanced \|  \|  \|  \| \| 73 \| AIDS Serodiagnosis/ \| 6740 \| Advanced \|  \|  \|  \| \| 74 \| hiv.mp,kw,kf. \| 345557 \| Advanced \|  \|  \|  \| \| 75 \| hiv??.mp,kw,kf. \| 347839 \| Advanced \|  \|  \|  \| \| 76 \| htlv*.mp,kw,kf. \| 13381 \| Advanced \|  \|  \|  \| \| 77 \| human t-cell leukemia virus*.mp,kw,kf. \| 3340 \| Advanced \|  \|  \|  \| \| 78 \| (acquired adj3 immun* adj3 (syndrome* or virus*)).mp,kw,kf. \| 92406 \| Advanced \|  \|  \|  \| \| 79 \| (human* adj3 immun* adj3 deficien* adj3 virus*).mp,kw,kf. \| 694 \| Advanced \|  \|  \|  \| \| 80 \| (human* adj3 immun* adj3 virus*).mp,kw,kf. \| 96631 \| Advanced \|  \|  \|  \| \| 81 \| (syndrome* adj3 lymphadenopath*).mp,kw,kf. \| 463 \| Advanced \|  \|  \|  \| \| 82 \| (lymphadenopath* adj3 (related or associated) adj3 (virus* or retrovirus* or lentivirus*)).mp,kw,kf. \| 321 \| Advanced \|  \|  \|  \| \| 83 \| slim disease.mp,kw,kf. \| 25 \| Advanced \|  \|  \|  \| \| 84 \| lav-htlv-iii.mp,kw,kf. \| 210 \| Advanced \|  \|  \|  \| \| 85 \| (sbl-6669 or sbl6669).mp,kw,kf. \| 25 \| Advanced \|  \|  \|  \| \| 86 \| (lav-2 or lav2).mp,kw,kf. \| 30 \| Advanced \|  \|  \|  \| \| 87 \| sbl6669.mp,kw,kf. \| 9 \| Advanced \|  \|  \|  \| \| 88 \| (acquired adj3 immun* adj3 deficien* adj3 syndrome*).mp,kw,kf. \| 5764 \| Advanced \|  \|  \|  \| \| 89 \| (aids adj10 (disease* or syndrome*)).mp,kw,kf. \| 31400 \| Advanced \|  \|  \|  \| \| 90 \| (aids adj3 associated adj3 (virus* or retrovirus* or lentivirus*)).mp,kw,kf. \| 170 \| Advanced \|  \|  \|  \| \| 91 \| (aids adj2 related).mp,kw,kf. \| 31218 \| Advanced \|  \|  \|  \| \| 92 \| or/69-91 \| 414927 \| Advanced \|  \|  \|  \| \| 93 \| Hemorrhagic Fever, Ebola/ \| 6031 \| Advanced \|  \|  \|  \| \| 94 \| Ebolavirus/ \| 3596 \| Advanced \|  \|  \|  \| \| 95 \| ebola*.mp,kw,kf. \| 8953 \| Advanced \|  \|  \|  \| \| 96 \| EVD.mp,kw,kf. \| 1868 \| Advanced \|  \|  \|  \| \| 97 \| EHF.mp,kw,kf. \| 596 \| Advanced \|  \|  \|  \| \| 98 \| txid?128951.mp,kw,kf. \| 0 \| Advanced \|  \|  \|  \| \| 99 \| txid?186536.mp,kw,kf. \| 0 \| Advanced \|  \|  \|  \| \| 100 \| or/93-99 \| 10379 \| Advanced \|  \|  \|  \| \| 101 \| Zika Virus Infection/ \| 5529 \| Advanced \|  \|  \|  \| \| 102 \| Zika Virus/ \| 4938 \| Advanced \|  \|  \|  \| \| 103 \| zika.mp,kw,kf. \| 7864 \| Advanced \|  \|  \|  \| \| 104 \| zikv.mp,kw,kf. \| 2966 \| Advanced \|  \|  \|  \| \| 105 \| txid?64320.mp,kw,kf. \| 0 \| Advanced \|  \|  \|  \| \| 106 \| or/101-105 \| 7880 \| Advanced \|  \|  \|  \| \| 107 \| West Nile Fever/ \| 4309 \| Advanced \|  \|  \|  \| \| 108 \| West Nile virus/ \| 4819 \| Advanced \|  \|  \|  \| \| 109 \| (west nile adj2 virus*).mp,kw,kf. \| 7069 \| Advanced \|  \|  \|  \| \| 110 \| (west nile adj2 flavivirus*).mp,kw,kf. \| 201 \| Advanced \|  \|  \|  \| \| 111 \| (west nile adj2 infection?).mp,kw,kf. \| 985 \| Advanced \|  \|  \|  \| \| 112 \| (west nile adj2 fever?).mp,kw,kf. \| 4569 \| Advanced \|  \|  \|  \| \| 113 \| (west nile adj2 encephalitis).mp,kw,kf. \| 546 \| Advanced \|  \|  \|  \| \| 114 \| (west nile adj2 meningitis).mp,kw,kf. \| 17 \| Advanced \|  \|  \|  \| \| 115 \| (west nile adj2 meningoencephalitis).mp,kw,kf. \| 39 \| Advanced \|  \|  \|  \| \| 116 \| (west nile adj2 myelitis).mp,kw,kf. \| 3 \| Advanced \|  \|  \|  \| \| 117 \| (egypt 101 adj2 virus*).mp,kw,kf. \| 7 \| Advanced \|  \|  \|  \| \| 118 \| (egypt 101 adj2 flavivirus*).mp,kw,kf. \| 0 \| Advanced \|  \|  \|  \| \| 119 \| (kunjin adj2 virus*).mp,kw,kf. \| 245 \| Advanced \|  \|  \|  \| \| 120 \| (kunjin adj2 flavivirus*).mp,kw,kf. \| 59 \| Advanced \|  \|  \|  \| \| 121 \| WNV.mp,kw,kf. \| 3691 \| Advanced \|  \|  \|  \| \| 122 \| txid?11077.mp,kw,kf. \| 0 \| Advanced \|  \|  \|  \| \| 123 \| txid?11082.mp,kw,kf. \| 0 \| Advanced \|  \|  \|  \| \| 124 \| or/107-123 \| 7862 \| Advanced \|  \|  \|  \| \| 125 \| 6 or 38 or 68 or 92 or 100 or 106 or 124 \| 709739 \| Advanced \|  \|  \|  \| \| 126 \| Financing, Government/ \| 21187 \| Advanced \|  \|  \|  \| \| 127 \| Public Assistance/ \| 2973 \| Advanced \|  \|  \|  \| \| 128 \| Food Assistance/ \| 1437 \| Advanced \|  \|  \|  \| \| 129 \| Medical Assistance/ \| 2545 \| Advanced \|  \|  \|  \| \| 130 \| Workers' Compensation/ \| 7664 \| Advanced \|  \|  \|  \| \| 131 \| exp Charities/ \| 3955 \| Advanced \|  \|  \|  \| \| 132 \| Child Welfare/ \| 22249 \| Advanced \|  \|  \|  \| \| 133 \| Aid to Families with Dependent Children/ \| 752 \| Advanced \|  \|  \|  \| \| 134 \| Foundations/ec [Economics] \| 688 \| Advanced \|  \|  \|  \| \| 135 \| Relief Work/ \| 4130 \| Advanced \|  \|  \|  \| \| 136 \| Sick Leave/ \| 6294 \| Advanced \|  \|  \|  \| \| 137 \| Family Leave/ \| 359 \| Advanced \|  \|  \|  \| \| 138 \| Parental Leave/ \| 763 \| Advanced \|  \|  \|  \| \| 139 \| Insurance, Disability/ \| 1531 \| Advanced \|  \|  \|  \| \| 140 \| Financial Support/ \| 3861 \| Advanced \|  \|  \|  \| \| 141 \| Social Security/ \| 7669 \| Advanced \|  \|  \|  \| \| 142 \| (economic adj2 (payment? or plan or plans or program* or intervention? or incentive?)).mp,kw,kf. \| 1958 \| Advanced \|  \|  \|  \| \| 143 \| (economic adj3 (relief or support*)).mp,kw,kf. \| 1389 \| Advanced \|  \|  \|  \| \| 144 \| (economic adj5 (assistance or benefit?)).mp,kw,kf. \| 6801 \| Advanced \|  \|  \|  \| \| 145 \| (financial adj5 (relief or assistance or support* or payment? or benefit? or plan or plans or incentive?)).mp,kw,kf. \| 17770 \| Advanced \|  \|  \|  \| \| 146 \| (financial adj3 (program* or aid)).mp,kw,kf. \| 1265 \| Advanced \|  \|  \|  \| \| 147 \| (income adj2 (relief or assistance or support* or payment? or benefit? or replacement?)).mp,kw,kf. \| 1054 \| Advanced \|  \|  \|  \| \| 148 \| (income adj3 (plan or plans or program*)).mp,kw,kf. \| 1539 \| Advanced \|  \|  \|  \| \| 149 \| (monetary adj2 (relief or assistance or support* or payment? or benefit? or plan or plans or replacement? or incentive?)).mp,kw,kf. \| 1912 \| Advanced \|  \|  \|  \| \| 150 \| (monetary adj5 program*).mp,kw,kf. \| 136 \| Advanced \|  \|  \|  \| \| 151 \| (government adj2 (relief or assistance or support* or payment? or benefit?)).mp,kw,kf. \| 2067 \| Advanced \|  \|  \|  \| \| 152 \| (government adj5 (subsid* or aid)).mp,kw,kf. \| 1089 \| Advanced \|  \|  \|  \| \| 153 \| (unemployment adj3 (relief or assistance or support* or payment? or benefit? or plan or plans or program* or replacement? or insurance)).mp,kw,kf. \| 547 \| Advanced \|  \|  \|  \| \| 154 \| (employment adj3 (relief or assistance or support* or payment? or benefit? or plan or plans or program* or replacement? or insurance)).mp,kw,kf. \| 3896 \| Advanced \|  \|  \|  \| \| 155 \| (social adj2 (relief or assistance or payment?)).mp,kw,kf. \| 1183 \| Advanced \|  \|  \|  \| \| 156 \| (economic adj2 impact* adj2 pay*).mp,kw,kf. \| 11 \| Advanced \|  \|  \|  \| \| 157 \| (federal adj2 aid).mp,kw,kf. \| 86 \| Advanced \|  \|  \|  \| \| 158 \| (federal adj2 allocation?).mp,kw,kf. \| 36 \| Advanced \|  \|  \|  \| \| 159 \| (public adj2 subsid*).mp,kw,kf. \| 280 \| Advanced \|  \|  \|  \| \| 160 \| (public adj2 assistance).mp,kw,kf. \| 3777 \| Advanced \|  \|  \|  \| \| 161 \| (food adj5 (security or insecurity) adj5 (support* or benefit? or plan or plans or program or aid)).mp,kw,kf. \| 453 \| Advanced \|  \|  \|  \| \| 162 \| (food adj2 assistance).mp,kw,kf. \| 1773 \| Advanced \|  \|  \|  \| \| 163 \| (food adj2 relief).mp,kw,kf. \| 68 \| Advanced \|  \|  \|  \| \| 164 \| (food adj2 aid).mp,kw,kf. \| 347 \| Advanced \|  \|  \|  \| \| 165 \| (food adj2 (fund? or funding)).mp,kw,kf. \| 58 \| Advanced \|  \|  \|  \| \| 166 \| (emergency adj3 medical adj3 assistance).mp,kw,kf. \| 146 \| Advanced \|  \|  \|  \| \| 167 \| child welfare.mp,kw,kf. \| 23408 \| Advanced \|  \|  \|  \| \| 168 \| (charity or charities).mp,kw,kf. \| 6544 \| Advanced \|  \|  \|  \| \| 169 \| almshous*.mp,kw,kf. \| 90 \| Advanced \|  \|  \|  \| \| 170 \| (("nonprofit" or "non-profit" or "not-for-profit") adj2 organi#ation*).mp,kw,kf. \| 4981 \| Advanced \|  \|  \|  \| \| 171 \| relief work?.mp,kw,kf. \| 4196 \| Advanced \|  \|  \|  \| \| 172 \| (humanitarian adj2 assistance).mp,kw,kf. \| 357 \| Advanced \|  \|  \|  \| \| 173 \| (sick adj4 leave?).mp,kw,kf. \| 8928 \| Advanced \|  \|  \|  \| \| 174 \| medical leave?.mp,kw,kf. \| 265 \| Advanced \|  \|  \|  \| \| 175 \| disability leave?.mp,kw,kf. \| 56 \| Advanced \|  \|  \|  \| \| 176 \| (sick adj2 day?).mp,kw,kf. \| 1184 \| Advanced \|  \|  \|  \| \| 177 \| illness day?.mp,kw,kf. \| 133 \| Advanced \|  \|  \|  \| \| 178 \| sick absence?.mp,kw,kf. \| 45 \| Advanced \|  \|  \|  \| \| 179 \| illness absence?.mp,kw,kf. \| 57 \| Advanced \|  \|  \|  \| \| 180 \| family leave?.mp,kw,kf. \| 442 \| Advanced \|  \|  \|  \| \| 181 \| parental leave?.mp,kw,kf. \| 902 \| Advanced \|  \|  \|  \| \| 182 \| ((childcare or child-care) adj2 benefit?).mp,kw,kf. \| 33 \| Advanced \|  \|  \|  \| \| 183 \| ((childcare or child-care) adj2 support*).mp,kw,kf. \| 183 \| Advanced \|  \|  \|  \| \| 184 \| ((childcare or child-care) adj2 assistance).mp,kw,kf. \| 55 \| Advanced \|  \|  \|  \| \| 185 \| ((childcare or child-care) adj2 welfare).mp,kw,kf. \| 11 \| Advanced \|  \|  \|  \| \| 186 \| (disabilit* adj2 insurance).mp,kw,kf. \| 2141 \| Advanced \|  \|  \|  \| \| 187 \| (worker?? adj2 compensation?).mp,kw,kf. \| 9048 \| Advanced \|  \|  \|  \| \| 188 \| ((workm#n* or workwom#n*) adj2 compensation?).mp,kw,kf. \| 1495 \| Advanced \|  \|  \|  \| \| 189 \| (covid* adj3 response adj3 (grant* or fund*)).mp,kw,kf. \| 6 \| Advanced \|  \|  \|  \| \| 190 \| (debt? adj2 relie*).mp,kw,kf. \| 48 \| Advanced \|  \|  \|  \| \| 191 \| (defer* adj2 payment?).mp,kw,kf. \| 8 \| Advanced \|  \|  \|  \| \| 192 \| emergency fund*3.mp,kw,kf. \| 147 \| Advanced \|  \|  \|  \| \| 193 \| emergency response benefit?.mp,kw,kf. \| 0 \| Advanced \|  \|  \|  \| \| 194 \| foreign aid.mp,kw,kf. \| 715 \| Advanced \|  \|  \|  \| \| 195 \| humanitarian aid.mp,kw,kf. \| 398 \| Advanced \|  \|  \|  \| \| 196 \| ((relief or recovery) adj2 fund*3).mp,kw,kf. \| 125 \| Advanced \|  \|  \|  \| \| 197 \| social protection program*.mp,kw,kf. \| 64 \| Advanced \|  \|  \|  \| \| 198 \| universal basic income?.mp,kw,kf. \| 16 \| Advanced \|  \|  \|  \| \| 199 \| (Coronavirus Aid Relief adj2 Economic Security Act).mp,kw,kf. \| 5 \| Advanced \|  \|  \|  \| \| 200 \| CARES act.mp,kw,kf. \| 17 \| Advanced \|  \|  \|  \| \| 201 \| (stimulus adj2 (cheque? or check? or payment? or money)).mp,kw,kf. \| 38 \| Advanced \|  \|  \|  \| \| 202 \| or/126-201 \| 134196 \| Advanced \|  \|  \|  \| \| 203 \| 125 and 202 \| 6931 \| Advanced \|  \|  \|  \| \| 204 \| animals/ not (animals/ and humans/) \| 4862009 \| Advanced \|  \|  \|  \| \| 205 \| 203 not 204 \| 6900 \| Advanced \|  \|  \|  \| \| 206 \| limit 205 to (clinical conference or comment or consensus development conference or consensus development conference, nih or editorial or letter) \| 340 \| Advanced \|  \|  \|  \| \| 207 \| 205 not 206 \| 6560 \| Advanced \|  \|  \|  \| \| 208 \| limit 207 to yr="2001 -Current" \| 4737 \| Advanced \|  \|  \|  \| \| 209 \| remove duplicates from 208 \| 4728 \| Advanced \|  \|  \|  \| |  |  |  |  |
| --- | --- | --- | --- | --- | --- | --- | --- | --- | --- | --- | --- | --- | --- | --- | --- | --- | --- | --- | --- | --- | --- | --- | --- | --- | --- | --- | --- | --- | --- | --- | --- | --- | --- | --- | --- | --- | --- | --- | --- | --- | --- | --- | --- | --- | --- | --- | --- | --- | --- | --- | --- | --- | --- | --- | --- | --- | --- | --- | --- | --- | --- | --- | --- | --- | --- | --- | --- | --- | --- | --- | --- | --- | --- | --- | --- | --- | --- | --- | --- | --- | --- | --- | --- | --- | --- | --- | --- | --- | --- | --- | --- | --- | --- | --- | --- | --- | --- | --- | --- | --- | --- | --- | --- | --- | --- | --- | --- | --- | --- | --- | --- | --- | --- | --- | --- | --- | --- | --- | --- | --- | --- | --- | --- | --- | --- | --- | --- | --- | --- | --- | --- | --- | --- | --- | --- | --- | --- | --- | --- | --- | --- | --- | --- | --- | --- | --- | --- | --- | --- | --- | --- | --- | --- | --- | --- | --- | --- | --- | --- | --- | --- | --- | --- | --- | --- | --- | --- | --- | --- | --- | --- | --- | --- | --- | --- | --- | --- | --- | --- | --- | --- | --- | --- | --- | --- | --- | --- | --- | --- | --- | --- | --- | --- | --- | --- | --- | --- | --- | --- | --- | --- | --- | --- | --- | --- | --- | --- | --- | --- | --- | --- | --- | --- | --- | --- | --- | --- | --- | --- | --- | --- | --- | --- | --- | --- | --- | --- | --- | --- | --- | --- | --- | --- | --- | --- | --- | --- | --- | --- | --- | --- | --- | --- | --- | --- | --- | --- | --- | --- | --- | --- | --- | --- | --- | --- | --- | --- | --- | --- | --- | --- | --- | --- | --- | --- | --- | --- | --- | --- | --- | --- | --- | --- | --- | --- | --- | --- | --- | --- | --- | --- | --- | --- | --- | --- | --- | --- | --- | --- | --- | --- | --- | --- | --- | --- | --- | --- | --- | --- | --- | --- | --- | --- | --- | --- | --- | --- | --- | --- | --- | --- | --- | --- | --- | --- | --- | --- | --- | --- | --- | --- | --- | --- | --- | --- | --- | --- | --- | --- | --- | --- | --- | --- | --- | --- | --- | --- | --- | --- | --- | --- | --- | --- | --- | --- | --- | --- | --- | --- | --- | --- | --- | --- | --- | --- | --- | --- | --- | --- | --- | --- | --- | --- | --- | --- | --- | --- | --- | --- | --- | --- | --- | --- | --- | --- | --- | --- | --- | --- | --- | --- | --- | --- | --- | --- | --- | --- | --- | --- | --- | --- | --- | --- | --- | --- | --- | --- | --- | --- | --- | --- | --- | --- | --- | --- | --- | --- | --- | --- | --- | --- | --- | --- | --- | --- | --- | --- | --- | --- | --- | --- | --- | --- | --- | --- | --- | --- | --- | --- | --- | --- | --- | --- | --- | --- | --- | --- | --- | --- | --- | --- | --- | --- | --- | --- | --- | --- | --- | --- | --- | --- | --- | --- | --- | --- | --- | --- | --- | --- | --- | --- | --- | --- | --- | --- | --- | --- | --- | --- | --- | --- | --- | --- | --- | --- | --- | --- | --- | --- | --- | --- | --- | --- | --- | --- | --- | --- | --- | --- | --- | --- | --- | --- | --- | --- | --- | --- | --- | --- | --- | --- | --- | --- | --- | --- | --- | --- | --- | --- | --- | --- | --- | --- | --- | --- | --- | --- | --- | --- | --- | --- | --- | --- | --- | --- | --- | --- | --- | --- | --- | --- | --- | --- | --- | --- | --- | --- | --- | --- | --- | --- | --- | --- | --- | --- | --- | --- | --- | --- | --- | --- | --- | --- | --- | --- | --- | --- | --- | --- | --- | --- | --- | --- | --- | --- | --- | --- | --- | --- | --- | --- | --- | --- | --- | --- | --- | --- | --- | --- | --- | --- | --- | --- | --- | --- | --- | --- | --- | --- | --- | --- | --- | --- | --- | --- | --- | --- | --- | --- | --- | --- | --- | --- | --- | --- | --- | --- | --- | --- | --- | --- | --- | --- | --- | --- | --- | --- | --- | --- | --- | --- | --- | --- | --- | --- | --- | --- | --- | --- | --- | --- | --- | --- | --- | --- | --- | --- | --- | --- | --- | --- | --- | --- | --- | --- | --- | --- | --- | --- | --- | --- | --- | --- | --- | --- | --- | --- | --- | --- | --- | --- | --- | --- | --- | --- | --- | --- | --- | --- | --- | --- | --- | --- | --- | --- | --- | --- | --- | --- | --- | --- | --- | --- | --- | --- | --- | --- | --- | --- | --- | --- | --- | --- | --- | --- | --- | --- | --- | --- | --- | --- | --- | --- | --- | --- | --- | --- | --- | --- | --- | --- | --- | --- | --- | --- | --- | --- | --- | --- | --- | --- | --- | --- | --- | --- | --- | --- | --- | --- | --- | --- | --- | --- | --- | --- | --- | --- | --- | --- | --- | --- | --- | --- | --- | --- | --- | --- | --- | --- | --- | --- | --- | --- | --- | --- | --- | --- | --- | --- | --- | --- | --- | --- | --- | --- | --- | --- | --- | --- | --- | --- | --- | --- | --- | --- | --- | --- | --- | --- | --- | --- | --- | --- | --- | --- | --- | --- | --- | --- | --- | --- | --- | --- | --- | --- | --- | --- | --- | --- | --- | --- | --- | --- | --- | --- | --- | --- | --- | --- | --- | --- | --- | --- | --- | --- | --- | --- | --- | --- | --- | --- | --- | --- | --- | --- | --- | --- | --- | --- | --- | --- | --- | --- | --- | --- | --- | --- | --- | --- | --- | --- | --- | --- | --- | --- | --- | --- | --- | --- | --- | --- | --- | --- | --- | --- | --- | --- | --- | --- | --- | --- | --- | --- | --- | --- | --- | --- | --- | --- | --- | --- | --- | --- | --- | --- | --- | --- | --- | --- | --- | --- | --- | --- | --- | --- | --- | --- | --- | --- | --- | --- | --- | --- | --- | --- | --- | --- | --- | --- | --- | --- | --- | --- | --- | --- | --- | --- | --- | --- | --- | --- | --- | --- | --- | --- | --- | --- | --- | --- | --- | --- | --- | --- | --- | --- | --- | --- | --- | --- | --- | --- | --- | --- | --- | --- | --- | --- | --- | --- | --- | --- | --- | --- | --- | --- | --- | --- | --- | --- | --- | --- | --- | --- | --- | --- | --- | --- | --- | --- | --- | --- | --- | --- | --- | --- | --- | --- | --- | --- | --- | --- | --- | --- | --- | --- | --- | --- | --- | --- | --- | --- | --- | --- | --- | --- | --- | --- | --- | --- | --- | --- | --- | --- | --- | --- | --- | --- | --- | --- | --- | --- | --- | --- | --- | --- | --- | --- | --- | --- | --- | --- | --- | --- | --- | --- | --- | --- | --- | --- | --- | --- | --- | --- | --- | --- | --- | --- | --- | --- | --- | --- | --- | --- | --- | --- | --- | --- | --- | --- | --- | --- | --- | --- | --- | --- | --- | --- | --- | --- | --- | --- | --- | --- | --- | --- | --- | --- | --- | --- | --- | --- | --- | --- | --- | --- | --- | --- | --- | --- | --- | --- | --- | --- | --- | --- | --- | --- | --- | --- | --- | --- | --- | --- | --- | --- | --- | --- | --- | --- | --- | --- | --- | --- | --- | --- | --- | --- | --- | --- | --- | --- | --- | --- | --- | --- | --- | --- | --- | --- | --- | --- | --- | --- | --- | --- | --- | --- | --- | --- | --- | --- | --- | --- | --- | --- | --- | --- | --- | --- | --- | --- | --- | --- | --- | --- | --- | --- | --- | --- | --- | --- | --- | --- | --- | --- | --- | --- | --- | --- | --- | --- | --- | --- | --- | --- | --- | --- | --- | --- | --- | --- | --- | --- | --- | --- | --- | --- | --- | --- | --- | --- | --- | --- | --- | --- | --- | --- | --- | --- | --- | --- | --- | --- | --- | --- | --- | --- | --- | --- | --- | --- | --- | --- | --- | --- | --- | --- | --- | --- | --- | --- | --- | --- | --- | --- | --- | --- | --- | --- | --- | --- | --- | --- | --- | --- | --- | --- | --- | --- | --- | --- | --- | --- | --- | --- | --- | --- | --- | --- | --- | --- | --- | --- | --- | --- | --- | --- | --- | --- | --- | --- | --- | --- | --- | --- | --- | --- | --- | --- | --- | --- | --- | --- | --- | --- | --- | --- | --- | --- | --- | --- | --- | --- | --- | --- | --- | --- | --- | --- | --- | --- | --- | --- | --- | --- | --- | --- | --- | --- | --- | --- | --- | --- | --- | --- | --- | --- | --- | --- | --- | --- | --- | --- | --- | --- | --- | --- | --- | --- | --- | --- | --- | --- | --- | --- | --- | --- | --- | --- | --- | --- | --- | --- | --- | --- | --- | --- | --- | --- | --- | --- | --- | --- | --- | --- | --- | --- | --- | --- | --- | --- | --- | --- | --- | --- | --- | --- | --- | --- | --- | --- | --- | --- | --- | --- | --- | --- | --- | --- | --- | --- | --- | --- | --- | --- | --- | --- | --- | --- | --- | --- | --- | --- | --- | --- | --- | --- | --- | --- | --- | --- | --- | --- | --- | --- | --- | --- | --- | --- | --- | --- | --- | --- | --- | --- | --- | --- | --- | --- | --- | --- | --- | --- | --- | --- | --- | --- | --- | --- | --- | --- | --- | --- | --- | --- | --- | --- | --- | --- | --- | --- | --- | --- | --- | --- | --- | --- | --- | --- | --- | --- | --- | --- | --- | --- | --- | --- | --- | --- | --- | --- | --- | --- | --- | --- | --- | --- | --- | --- | --- | --- | --- | --- | --- | --- | --- | --- | --- | --- | --- | --- | --- | --- | --- | --- | --- | --- | --- | --- | --- | --- | --- | --- | --- | --- | --- | --- | --- | --- | --- | --- | --- | --- | --- | --- | --- | --- | --- | --- | --- | --- | --- |
|  |  |  |  |  |
