# Supplementary material for: Health and non-health benefits and equity impacts of individual-level economic relief programs during epidemics/pandemics in high income settings: a scoping review
Source: BMC Public Health. 2024 Aug 5;24:2106. doi: 10.1186/s12889-024-19493-8 (PMC11299345; doi:10.1186/s12889-024-19493-8)
Supplement: Supplementary file 2 — Supplementary Material 2. Updated Ovid Medline search strategy from January 1, 2021, April 3, 2023. [file 12889_2024_19493_MOESM2_ESM.docx]

**Updated Ovid MEDLINE search strategy <1946 to March 31, 2023>**

| **#** | **Searches** | **Results** | **Type** |  |  |  |  |  |  |
| --- | --- | --- | --- | --- | --- | --- | --- | --- | --- |
|  | | | |  |  |  |  |  |  |
| 1 | Epidemics/ | 13711 | Advanced |  |  |  |  |  |  |
| 2 | Pandemics/ | 111032 | Advanced |  |  |  |  |  |  |
| 3 | epidemic?.mp,kw,kf. | 119103 | Advanced |  |  |  |  |  |  |
| 4 | pandemic?.mp,kw,kf. | 160572 | Advanced |  |  |  |  |  |  |
| 5 | ((infectious or communicable) adj3 disease? adj3 outbreak*).mp,kw,kf. | 2442 | Advanced |  |  |  |  |  |  |
| 6 | or/1-5 | 269208 | Advanced |  |  |  |  |  |  |
| 7 | exp Coronavirus/ | 164572 | Advanced |  |  |  |  |  |  |
| 8 | exp Coronavirus Infections/ | 228606 | Advanced |  |  |  |  |  |  |
| 9 | Severe Acute Respiratory Syndrome/ | 5727 | Advanced |  |  |  |  |  |  |
| 10 | SARS-CoV-2/ | 150891 | Advanced |  |  |  |  |  |  |
| 11 | SARS Virus/ | 4176 | Advanced |  |  |  |  |  |  |
| 12 | Middle East Respiratory Syndrome Coronavirus/ | 1986 | Advanced |  |  |  |  |  |  |
| 13 | COVID-19/ | 217137 | Advanced |  |  |  |  |  |  |
| 14 | (coronavirus* or corona virus*).mp,kw,kf. | 117861 | Advanced |  |  |  |  |  |  |
| 15 | (coronavirinae* or corona virinae*).mp,kw,kf. | 34 | Advanced |  |  |  |  |  |  |
| 16 | (txid1898672 or txid 1898672).mp,kw,kf. | 0 | Advanced |  |  |  |  |  |  |
| 17 | alphacoronavirus*.mp,kw,kf. | 365 | Advanced |  |  |  |  |  |  |
| 18 | (txid693996 or txid 693996).mp,kw,kf. | 0 | Advanced |  |  |  |  |  |  |
| 19 | betacoronavirus*.mp,kw,kf. | 33894 | Advanced |  |  |  |  |  |  |
| 20 | (txid694002 or txid 694002).mp,kw,kf. | 0 | Advanced |  |  |  |  |  |  |
| 21 | covid*.mp,kw,kf. | 228037 | Advanced |  |  |  |  |  |  |
| 22 | ncovid*.mp,kw,kf. | 31 | Advanced |  |  |  |  |  |  |
| 23 | HCoV*.mp,kw,kf. | 1236 | Advanced |  |  |  |  |  |  |
| 24 | NCoV????.mp,kw,kf. | 3483 | Advanced |  |  |  |  |  |  |
| 25 | (CoV2* or CoV-2*).mp,kw,kf. | 161207 | Advanced |  |  |  |  |  |  |
| 26 | (CoV19 or CoV-19).mp,kw,kf. | 173 | Advanced |  |  |  |  |  |  |
| 27 | (CoV2019 or CoV-2019).mp,kw,kf. | 20 | Advanced |  |  |  |  |  |  |
| 28 | (2019nCoV or 2019-nCoV).mp,kw,kf. | 2228 | Advanced |  |  |  |  |  |  |
| 29 | (SARS-CoV* or SARSCoV*).mp,kw,kf. | 163604 | Advanced |  |  |  |  |  |  |
| 30 | (2019nCoV* or 2019-nCoV*).mp,kw,kf. | 2247 | Advanced |  |  |  |  |  |  |
| 31 | (SARS or SARSr).mp,kw,kf. | 169935 | Advanced |  |  |  |  |  |  |
| 32 | (severe acute respiratory adj2 syndrome*).mp,kw,kf. | 34228 | Advanced |  |  |  |  |  |  |
| 33 | (txid694009 or txid 694009).mp,kw,kf. | 0 | Advanced |  |  |  |  |  |  |
| 34 | (middle eastern respiratory adj2 syndrome*).mp,kw,kf. | 75 | Advanced |  |  |  |  |  |  |
| 35 | MERS.mp,kw,kf. | 6146 | Advanced |  |  |  |  |  |  |
| 36 | (MERSCoV* or MERS-Cov*).mp,kw,kf. | 2817 | Advanced |  |  |  |  |  |  |
| 37 | (txid1335626 or txid 1335626).mp,kw,kf. | 0 | Advanced |  |  |  |  |  |  |
| 38 | or/7-37 | 256003 | Advanced |  |  |  |  |  |  |
| 39 | Influenza A virus/ | 22796 | Advanced |  |  |  |  |  |  |
| 40 | Influenza A Virus, H1N1 Subtype/ | 17390 | Advanced |  |  |  |  |  |  |
| 41 | Influenza A Virus, H2N2 Subtype/ | 235 | Advanced |  |  |  |  |  |  |
| 42 | Influenza A Virus, H3N2 Subtype/ | 5010 | Advanced |  |  |  |  |  |  |
| 43 | Influenza Pandemic, 1918-1919/ | 258 | Advanced |  |  |  |  |  |  |
| 44 | influenza A.mp,kw,kf. | 55737 | Advanced |  |  |  |  |  |  |
| 45 | flu A.mp,kw,kf. | 447 | Advanced |  |  |  |  |  |  |
| 46 | fowl plague virus*.mp,kw,kf. | 379 | Advanced |  |  |  |  |  |  |
| 47 | grippe.mp,kw,kf. | 1290 | Advanced |  |  |  |  |  |  |
| 48 | pestis galli myxovirus*.mp,kw,kf. | 0 | Advanced |  |  |  |  |  |  |
| 49 | orthomyxovirus*.mp,kw,kf. | 355 | Advanced |  |  |  |  |  |  |
| 50 | (txid11320 or txid 11320).mp,kw,kf. | 0 | Advanced |  |  |  |  |  |  |
| 51 | H1N1.mp,kw,kf. | 21747 | Advanced |  |  |  |  |  |  |
| 52 | (spanish adj2 (influenza?? or flu)).mp,kw,kf. | 535 | Advanced |  |  |  |  |  |  |
| 53 | (("1918" or "1919") adj2 (influenza?? or flu)).mp,kw,kf. | 1018 | Advanced |  |  |  |  |  |  |
| 54 | (russian* adj2 (influenza?? or flu)).mp,kw,kf. | 43 | Advanced |  |  |  |  |  |  |
| 55 | ("1977" adj2 (influenza?? or flu)).mp,kw,kf. | 35 | Advanced |  |  |  |  |  |  |
| 56 | (swine adj2 (influenza?? or flu)).mp,kw,kf. | 3026 | Advanced |  |  |  |  |  |  |
| 57 | ("2009" adj2 (influenza?? or flu)).mp,kw,kf. | 3531 | Advanced |  |  |  |  |  |  |
| 58 | (txid114727 or txid 114727).mp,kw,kf. | 0 | Advanced |  |  |  |  |  |  |
| 59 | H2N2.mp,kw,kf. | 678 | Advanced |  |  |  |  |  |  |
| 60 | (asian adj2 (influenza?? or flu)).mp,kw,kf. | 603 | Advanced |  |  |  |  |  |  |
| 61 | (("1957" or "1958") adj2 (influenza?? or flu)).mp,kw,kf. | 158 | Advanced |  |  |  |  |  |  |
| 62 | H3N2.mp,kw,kf. | 8229 | Advanced |  |  |  |  |  |  |
| 63 | (hong kong adj2 (influenza?? or flu)).mp,kw,kf. | 560 | Advanced |  |  |  |  |  |  |
| 64 | (("1968" or "1969") adj2 (influenza?? or flu)).mp,kw,kf. | 111 | Advanced |  |  |  |  |  |  |
| 65 | (fujian adj2 (influenza?? or flu)).mp,kw,kf. | 16 | Advanced |  |  |  |  |  |  |
| 66 | (("2003" or "2004") adj2 (influenza?? or flu)).mp,kw,kf. | 387 | Advanced |  |  |  |  |  |  |
| 67 | (txid119210 or txid 119210).mp,kw,kf. | 0 | Advanced |  |  |  |  |  |  |
| 68 | or/39-67 | 61020 | Advanced |  |  |  |  |  |  |
| 69 | Hemorrhagic Fever, Ebola/ | 6588 | Advanced |  |  |  |  |  |  |
| 70 | Ebolavirus/ | 3948 | Advanced |  |  |  |  |  |  |
| 71 | ebola*.mp,kw,kf. | 9867 | Advanced |  |  |  |  |  |  |
| 72 | EVD.mp,kw,kf. | 2126 | Advanced |  |  |  |  |  |  |
| 73 | EHF.mp,kw,kf. | 664 | Advanced |  |  |  |  |  |  |
| 74 | txid?128951.mp,kw,kf. | 0 | Advanced |  |  |  |  |  |  |
| 75 | txid?186536.mp,kw,kf. | 0 | Advanced |  |  |  |  |  |  |
| 76 | or/69-75 | 11471 | Advanced |  |  |  |  |  |  |
| 77 | Zika Virus Infection/ | 6891 | Advanced |  |  |  |  |  |  |
| 78 | Zika Virus/ | 6320 | Advanced |  |  |  |  |  |  |
| 79 | zika.mp,kw,kf. | 9507 | Advanced |  |  |  |  |  |  |
| 80 | zikv.mp,kw,kf. | 3744 | Advanced |  |  |  |  |  |  |
| 81 | txid?64320.mp,kw,kf. | 0 | Advanced |  |  |  |  |  |  |
| 82 | or/77-81 | 9529 | Advanced |  |  |  |  |  |  |
| 83 | West Nile Fever/ | 4557 | Advanced |  |  |  |  |  |  |
| 84 | West Nile virus/ | 5127 | Advanced |  |  |  |  |  |  |
| 85 | (west nile adj2 virus*).mp,kw,kf. | 7520 | Advanced |  |  |  |  |  |  |
| 86 | (west nile adj2 flavivirus*).mp,kw,kf. | 217 | Advanced |  |  |  |  |  |  |
| 87 | (west nile adj2 infection?).mp,kw,kf. | 1046 | Advanced |  |  |  |  |  |  |
| 88 | (west nile adj2 fever?).mp,kw,kf. | 4843 | Advanced |  |  |  |  |  |  |
| 89 | (west nile adj2 encephalitis).mp,kw,kf. | 594 | Advanced |  |  |  |  |  |  |
| 90 | (west nile adj2 meningitis).mp,kw,kf. | 17 | Advanced |  |  |  |  |  |  |
| 91 | (west nile adj2 meningoencephalitis).mp,kw,kf. | 41 | Advanced |  |  |  |  |  |  |
| 92 | (west nile adj2 myelitis).mp,kw,kf. | 3 | Advanced |  |  |  |  |  |  |
| 93 | (egypt 101 adj2 virus*).mp,kw,kf. | 7 | Advanced |  |  |  |  |  |  |
| 94 | (egypt 101 adj2 flavivirus*).mp,kw,kf. | 0 | Advanced |  |  |  |  |  |  |
| 95 | (kunjin adj2 virus*).mp,kw,kf. | 251 | Advanced |  |  |  |  |  |  |
| 96 | (kunjin adj2 flavivirus*).mp,kw,kf. | 59 | Advanced |  |  |  |  |  |  |
| 97 | WNV.mp,kw,kf. | 3979 | Advanced |  |  |  |  |  |  |
| 98 | txid?11077.mp,kw,kf. | 0 | Advanced |  |  |  |  |  |  |
| 99 | txid?11082.mp,kw,kf. | 0 | Advanced |  |  |  |  |  |  |
| 100 | or/83-99 | 8365 | Advanced |  |  |  |  |  |  |
| 101 | 6 or 38 or 68 or 76 or 82 or 100 | 449851 | Advanced |  |  |  |  |  |  |
| 102 | Financing, Government/ | 21365 | Advanced |  |  |  |  |  |  |
| 103 | Public Assistance/ | 3012 | Advanced |  |  |  |  |  |  |
| 104 | Food Assistance/ | 1784 | Advanced |  |  |  |  |  |  |
| 105 | Medical Assistance/ | 2776 | Advanced |  |  |  |  |  |  |
| 106 | Workers' Compensation/ | 7823 | Advanced |  |  |  |  |  |  |
| 107 | exp Charities/ | 4043 | Advanced |  |  |  |  |  |  |
| 108 | Child Welfare/ | 22532 | Advanced |  |  |  |  |  |  |
| 109 | Aid to Families with Dependent Children/ | 753 | Advanced |  |  |  |  |  |  |
| 110 | Foundations/ec [Economics] | 690 | Advanced |  |  |  |  |  |  |
| 111 | Relief Work/ | 4242 | Advanced |  |  |  |  |  |  |
| 112 | Sick Leave/ | 6705 | Advanced |  |  |  |  |  |  |
| 113 | Family Leave/ | 375 | Advanced |  |  |  |  |  |  |
| 114 | Parental Leave/ | 871 | Advanced |  |  |  |  |  |  |
| 115 | Insurance, Disability/ | 1564 | Advanced |  |  |  |  |  |  |
| 116 | Financial Support/ | 3915 | Advanced |  |  |  |  |  |  |
| 117 | Social Security/ | 7824 | Advanced |  |  |  |  |  |  |
| 118 | (economic adj2 (payment? or plan or plans or program* or intervention? or incentive?)).mp,kw,kf. | 2201 | Advanced |  |  |  |  |  |  |
| 119 | (economic adj3 (relief or support*)).mp,kw,kf. | 1719 | Advanced |  |  |  |  |  |  |
| 120 | (economic adj5 (assistance or benefit?)).mp,kw,kf. | 8107 | Advanced |  |  |  |  |  |  |
| 121 | (financial adj5 (relief or assistance or support* or payment? or benefit? or plan or plans or incentive?)).mp,kw,kf. | 20022 | Advanced |  |  |  |  |  |  |
| 122 | (financial adj3 (program* or aid)).mp,kw,kf. | 1469 | Advanced |  |  |  |  |  |  |
| 123 | (income adj2 (relief or assistance or support* or payment? or benefit? or replacement?)).mp,kw,kf. | 1233 | Advanced |  |  |  |  |  |  |
| 124 | (income adj3 (plan or plans or program*)).mp,kw,kf. | 1712 | Advanced |  |  |  |  |  |  |
| 125 | (monetary adj2 (relief or assistance or support* or payment? or benefit? or plan or plans or replacement? or incentive?)).mp,kw,kf. | 2335 | Advanced |  |  |  |  |  |  |
| 126 | (monetary adj5 program*).mp,kw,kf. | 148 | Advanced |  |  |  |  |  |  |
| 127 | (government adj2 (relief or assistance or support* or payment? or benefit?)).mp,kw,kf. | 2465 | Advanced |  |  |  |  |  |  |
| 128 | (government adj5 (subsid* or aid)).mp,kw,kf. | 1327 | Advanced |  |  |  |  |  |  |
| 129 | (unemployment adj3 (relief or assistance or support* or payment? or benefit? or plan or plans or program* or replacement? or insurance)).mp,kw,kf. | 650 | Advanced |  |  |  |  |  |  |
| 130 | (employment adj3 (relief or assistance or support* or payment? or benefit? or plan or plans or program* or replacement? or insurance)).mp,kw,kf. | 4375 | Advanced |  |  |  |  |  |  |
| 131 | (social adj2 (relief or assistance or payment?)).mp,kw,kf. | 1361 | Advanced |  |  |  |  |  |  |
| 132 | (economic adj2 impact* adj2 pay*).mp,kw,kf. | 15 | Advanced |  |  |  |  |  |  |
| 133 | (federal adj2 aid).mp,kw,kf. | 88 | Advanced |  |  |  |  |  |  |
| 134 | (federal adj2 allocation?).mp,kw,kf. | 41 | Advanced |  |  |  |  |  |  |
| 135 | (public adj2 subsid*).mp,kw,kf. | 317 | Advanced |  |  |  |  |  |  |
| 136 | (public adj2 assistance).mp,kw,kf. | 3893 | Advanced |  |  |  |  |  |  |
| 137 | (food adj5 (security or insecurity) adj5 (support* or benefit? or plan or plans or program or aid)).mp,kw,kf. | 628 | Advanced |  |  |  |  |  |  |
| 138 | (food adj2 assistance).mp,kw,kf. | 2197 | Advanced |  |  |  |  |  |  |
| 139 | (food adj2 relief).mp,kw,kf. | 78 | Advanced |  |  |  |  |  |  |
| 140 | (food adj2 aid).mp,kw,kf. | 388 | Advanced |  |  |  |  |  |  |
| 141 | (food adj2 (fund? or funding)).mp,kw,kf. | 71 | Advanced |  |  |  |  |  |  |
| 142 | (emergency adj3 medical adj3 assistance).mp,kw,kf. | 156 | Advanced |  |  |  |  |  |  |
| 143 | child welfare.mp,kw,kf. | 23859 | Advanced |  |  |  |  |  |  |
| 144 | (charity or charities).mp,kw,kf. | 6978 | Advanced |  |  |  |  |  |  |
| 145 | almshous*.mp,kw,kf. | 91 | Advanced |  |  |  |  |  |  |
| 146 | (("nonprofit" or "non-profit" or "not-for-profit") adj2 organi#ation*).mp,kw,kf. | 5319 | Advanced |  |  |  |  |  |  |
| 147 | relief work?.mp,kw,kf. | 4317 | Advanced |  |  |  |  |  |  |
| 148 | (humanitarian adj2 assistance).mp,kw,kf. | 406 | Advanced |  |  |  |  |  |  |
| 149 | (sick adj4 leave?).mp,kw,kf. | 9670 | Advanced |  |  |  |  |  |  |
| 150 | medical leave?.mp,kw,kf. | 310 | Advanced |  |  |  |  |  |  |
| 151 | disability leave?.mp,kw,kf. | 64 | Advanced |  |  |  |  |  |  |
| 152 | (sick adj2 day?).mp,kw,kf. | 1313 | Advanced |  |  |  |  |  |  |
| 153 | illness day?.mp,kw,kf. | 147 | Advanced |  |  |  |  |  |  |
| 154 | sick absence?.mp,kw,kf. | 48 | Advanced |  |  |  |  |  |  |
| 155 | illness absence?.mp,kw,kf. | 59 | Advanced |  |  |  |  |  |  |
| 156 | family leave?.mp,kw,kf. | 482 | Advanced |  |  |  |  |  |  |
| 157 | parental leave?.mp,kw,kf. | 1057 | Advanced |  |  |  |  |  |  |
| 158 | ((childcare or child-care) adj2 benefit?).mp,kw,kf. | 38 | Advanced |  |  |  |  |  |  |
| 159 | ((childcare or child-care) adj2 support*).mp,kw,kf. | 226 | Advanced |  |  |  |  |  |  |
| 160 | ((childcare or child-care) adj2 assistance).mp,kw,kf. | 62 | Advanced |  |  |  |  |  |  |
| 161 | ((childcare or child-care) adj2 welfare).mp,kw,kf. | 12 | Advanced |  |  |  |  |  |  |
| 162 | (disabilit* adj2 insurance).mp,kw,kf. | 2224 | Advanced |  |  |  |  |  |  |
| 163 | (worker?? adj2 compensation?).mp,kw,kf. | 9303 | Advanced |  |  |  |  |  |  |
| 164 | ((workm#n* or workwom#n*) adj2 compensation?).mp,kw,kf. | 1497 | Advanced |  |  |  |  |  |  |
| 165 | (covid* adj3 response adj3 (grant* or fund*)).mp,kw,kf. | 17 | Advanced |  |  |  |  |  |  |
| 166 | (debt? adj2 relie*).mp,kw,kf. | 54 | Advanced |  |  |  |  |  |  |
| 167 | (defer* adj2 payment?).mp,kw,kf. | 7 | Advanced |  |  |  |  |  |  |
| 168 | emergency fund*3.mp,kw,kf. | 174 | Advanced |  |  |  |  |  |  |
| 169 | emergency response benefit?.mp,kw,kf. | 1 | Advanced |  |  |  |  |  |  |
| 170 | foreign aid.mp,kw,kf. | 737 | Advanced |  |  |  |  |  |  |
| 171 | humanitarian aid.mp,kw,kf. | 479 | Advanced |  |  |  |  |  |  |
| 172 | ((relief or recovery) adj2 fund*3).mp,kw,kf. | 154 | Advanced |  |  |  |  |  |  |
| 173 | social protection program*.mp,kw,kf. | 85 | Advanced |  |  |  |  |  |  |
| 174 | universal basic income?.mp,kw,kf. | 29 | Advanced |  |  |  |  |  |  |
| 175 | (Coronavirus Aid Relief adj2 Economic Security Act).mp,kw,kf. | 9 | Advanced |  |  |  |  |  |  |
| 176 | CARES act.mp,kw,kf. | 32 | Advanced |  |  |  |  |  |  |
| 177 | (stimulus adj2 (cheque? or check? or payment? or money)).mp,kw,kf. | 47 | Advanced |  |  |  |  |  |  |
| 178 | or/102-177 | 144172 | Advanced |  |  |  |  |  |  |
| 179 | 101 and 178 | 4016 | Advanced |  |  |  |  |  |  |
| 180 | animals/ not (animals/ and humans/) | 5073908 | Advanced |  |  |  |  |  |  |
| 181 | 179 not 180 | 3982 | Advanced |  |  |  |  |  |  |
| 182 | limit 181 to (clinical conference or comment or consensus development conference or consensus development conference, nih or editorial or letter) | 213 | Advanced |  |  |  |  |  |  |
| 183 | 181 not 182 | 3769 | Advanced |  |  |  |  |  |  |
| 184 | remove duplicates from 183 | 3729 | Advanced |  |  |  |  |  |  |
| 185 | 202110*.ed. | 149493 | Advanced |  |  |  |  |  |  |
| 186 | 202111*.ed. | 133498 | Advanced |  |  |  |  |  |  |
| 187 | 202112*.ed. | 110002 | Advanced |  |  |  |  |  |  |
| 188 | 2022*.ed. | 1234119 | Advanced |  |  |  |  |  |  |
| 189 | 2023*.ed. | 279246 | Advanced |  |  |  |  |  |  |
| 190 | 185 or 186 or 187 or 188 or 189 | 1906358 | Advanced |  |  |  |  |  |  |
| 191 | 184 and 190 | 1337 | Advanced |  |  |  |  |  |  |
|  | | |  | | | |  |  |  |

**Meaning of some abbreviations used in the MEDLINE search strategy**

*exp- exploded; mp- multipurpose; kw- keywords; adj- adjacent; yr-year; tx-text word;*- truncated*

An extensive list of the meaning of abbreviations used in MEDLINE search engine can be found through this link: <https://ospguides.ovid.com/OSPguides/medline.htm>
