# Supplementary material for: Health and non-health benefits and equity impacts of individual-level economic relief programs during epidemics/pandemics in high income settings: a scoping review
Source: BMC Public Health. 2024 Aug 5;24:2106. doi: 10.1186/s12889-024-19493-8 (PMC11299345; doi:10.1186/s12889-024-19493-8)
Supplement: Supplementary file 3 — Supplementary Material 3. A comprehensive dataset of the outcomes extracted from the 50 eligible studies. [file 12889_2024_19493_MOESM3_ESM.docx]

| **Author, year** | **Study country** | **Study design**  Condensed data extraction file showing the outcomes extracted from eligible studies | **Economic relief program** | **Eligible population** | **How and to what extent do the EE program impact on public health measures** | **How and to what extent do the EE+ Public health measure effect impact on health** | **How and to what extent do the EE program impact on health** | **Do health benefits associated with the EE program differ across demographic groups (age, gender, race, /ethnicity/ culture/ language, and occupation)? If so, how?** | **Do health benefits associated with the EE program differ across social groups (e.g., socioeconomic status, and level of education)? If so, how?** | **Do health benefits associated with the EE program differ across jurisdictions (e.g., indigenous setting, poverty-stricken setting, urban vs rural)** | **What are the non-health outcomes reported in the studies ?** | |
| --- | --- | --- | --- | --- | --- | --- | --- | --- | --- | --- | --- | --- |
| Persaud, 2021 | Canada | randomized controlled trial | cash transfer | Patients reporting trouble affording basic necessities due to disruptions related to COVID-19. | no difference between groups in the number of close contacts outside of the household | cash transfer did not lower the symptom count up to 2 weeks; no difference in the percentage of patients who met the clinical definition of probable COVID-19. General health did not differ between groups after 2 weeks (60% vs 63%). | Self-reported health: General health did not differ between groups after 2 weeks (60%vs 63%). | In prespecified subgroup analyses, there was no difference between females and males , but cash transfers reduced the symptom count in those aged 50 years or older | NR | NR | Food insecurity was not reduced by cash transfers (80%vs 71%). | |
| Pichler, 2020 | USA | survey | expanded paid sick leave | populations eligible for state or federal COVID-19 paid sick leave | all models show statistically sig nificant decreases in the number of reported new  COVID-19 cases for states whose workers gained access to paid sick leave as a result of FFCRA . | Relative to the mean number of new cases for the post- FFCRA period for the control group, which is 749, a decrease of 417 cases in the preferred specification which translates  into a decrease of 56 percent | NR | NR | NR | NR | NR | |
| Tsai, 2020 | USA | survey | cash transfer | low and middle-income U.S. adults who received economic impact payment | NR | NR | EIP recipients were more likely to have tested positive or been untested for COVID-19, and to have been diagnosed before with schizophrenia-spectrum disorder and posttraumatic stress-disorder than those who did not receive the EIP; | SNR | Correlational analysis revealed that screening positive for current Major Depressive Disorder, Generalized Anxiety Disorder, COVID-19 era-related stress, and Alcohol Use Disorder were each strongly and significantly associated with problems paying for rent/mortgage (r= .16-.25)., utilities (r= .15-.26), food (r= .20-.37), transportation (r= .13-.23), clothing (r= .10-.22), and medical care (r= .07-.18) in the past month. | NR | EIP receipt was associated with fewer problems paying daily expenses | |
| Berkowitz, 2020 | USA | survey | unemployment assistance | working age adults with ongoing pandemic-related income disruption | NR | NR | consistently found that receiving UI benefits was associated with lower risk for unmet health-related social needs, and depressive and anxiety symptoms:depressive symptoms was 6.04 percentage points greater (95% CI 3.10 to 8.97,  p < 0.0001, q = 0.0002), and of anxiety symptoms was 5.82 percentage points greater (95%  CI 2.90 to 8.75, p < 0.0001, q = 0.0002). | NR | NR | NR | food insufficiency in those who received UI benefits compared with those who did not was 5.01 percentage points lower (95%CI 6.51 lower to 3.51 lower, p <0.0001, q = <0.0001). | |
| Marinescu, 2021 | USA | survey | unemployment assistance | populations eligible for COVID-19 unemployment benefits | NR | NR | NR | NR | NR | NR | 10% increase in unemployment benefits caused a 3.6% decline in job applications, but did not decrease vacancy creation; hence, FPUC increased labor market tightness (vacancies/applications). | |
| Kim, 2020 | South Korea | survey | cash transfer | individuals entitled to the consumption vouchers from the central government and municipal or regional governments | NR | NR | NR | NR | NR |  | Household consumption spending (to relieve economic distress): the voucher scheme successfully increased consumption spending among 36% of the households, and total spending to a lesser extent (29%), but the magnitude of the spending increase did not exceed the value of the transfers, leading to only 11% of households having a smaller amount of savings. | |
| Bienvenido-Huertas, 2021 | Spain | simulation | mixed | forloughed workers and low income earners eligible for unemployment benefits | NR | NR | NR | NR | NR | NR | unemployment aids can contribute to alleviating energy poverty, especially if the unemployed individual worked in a poorly-paying job or for just a few hours. | |
| Han, 2020 | USA | simulation | cash transfer | individuals with income less than $75,000, single parents with income below $112,500, and married couples with income less than $150,000. | NR | NR | NR | NR | NR | NR | Our results indicate that at the start of the pandemic, government policy effectively countered its effects on incomes, leading poverty to fall and low percentiles of income to rise across a range of demographic groups and geographies. | |
| Karger, 2021 | USA | simulation | cash transfer | recipients of Covid-19 Economic Impact Payments in anonymized transaction-level bank account data from Facteus. | NR | NR | NR | NR | NR | NR | In the two weeks following a $1,200 stimulus payment in April 2020, consumers increased spending by $546, implying a marginal propensity to consume of 46%. | |
| Fang, 2020 | USA | simulation | unemployment assistance | simulated young workers, young out of labor force, and senior adults | Paper assessed no EE, only shutdown, UI +shutdown | By lowering employment in the contact sector, both the shutdown and CARES UI policies reduce the peak infection and shift the infection. I shutdown alone. | NR | NR | CARES UI policies improved welfare for working population by 0.72% increase | NR | CARES UI policies raised average unemployment by 3.8 percentage points but also reduce cumulative deaths by 4.9 percent. | |
| Lee, 2021 | South Korea | epi model | incentive for testing | all population in simulated South Korea population | Assumed to increase testing | when financial incentives are provided, there are changes(INCREASE) in the proportion of confirmed patients out of unidentified infected people in the usceptible-unidentified infected-confirmed (SUC) epi model. | na | nr | nr | nr | The larger the incentive budget is, the faster the epidemic will end. | |
| Evangelist, 2022 | USA | survey | unemployment assistance | US credit and debit card purchases data for US population | NR | NR | NR | NR | NR | NR | Health care services spending declined by 1% for each percentage point increase in the unemployment insurance claims | |
| Stiemele, 2021 | USA | survey | food assistance | Families with children in 16 school districts in Pennsylvania | NR | NR | NR | NR | NR | NR | the proportion of SNAP users who skipped meals declined over time, decreasing by about 0.08 percentage points per day | |
| Schneider, 2021 | USA | quasi experimental | expanded paid sick leave | adults employed at large retail or food service firms | significant 15-percentage-point reduction in the share of Olive Garden workers who reported working while sick in the prior month. | NR | NR | 21.2% reduction of presenteeism while sick was observed in those workers with at least two years of job tenure, who would have had time to accrue paid sick leave. | NR | NR | Although workers at comparison firms saw no real change in their access to paid sick leave, the share of Olive Garden workers reporting access increased substantially and significantly from 23% in spring 2019 to 66% in spring 2020. | |
| Raifman, 2021 | USA | cohort | unemployment assistance | inidividuals living in households earning less than $75000 in the last 12 months and lost employment at some point during the April to November study period. | | | | | | | receipt of unemployment insurance was associated with a 4.3 (95%CI, 1.8-6.9) percentage point decrease in food insecurity (a 35.0% relative reduction) and a 5.7 (95%CI, 3.0-8.4) percentage point decrease in eating less due to financial constraints (a 47.8% relative reduction). | |
| Kubota, 2021 | Japan | survey | cash transfer | residents/households in Japan from January 2019 to August 2020. | NR | NR | NR | NR | NR | NR | there was an immediate jump in spending during the week of payments, followed by moderately elevated levels of spending that persist for more than one month after payments are received. | |
| Fang, 2020 | USA | survey | food assistance | adults with incomes less than 200% of the Federal Poverty Line. The subpopulation included SNAP eligible, those with lost their job, those with children or not, those employed and race (black, hispanic white) | NR | NR | NR | NR | NA | NA | receipt of unemployment benefits is negatively associated with food insecurity among those who lost a job due to the pandemic, households with children, and Black households. Receipt of economic stimulus payments was also negatively associated with food insecurity | |
| Ikeda 2021 | Japan | survey | mixed | Japanese households with children under the third grade of junior high school i | NR | NR | the Special Cash Payment was associated with better HRQOL utility score of the coefficient (95% CI): 0.05 (0.03 to 0.08). the Temporary Special Benefit for Child-raising Households was not associated with HRQOL utility score(95%CI): 0.00 (-0.02 to 0.03). | NR | NR | NR | NR |  |
| Martin, 2020 | USA | simulation | mixed | all population in the US | NR | NR | NR | NR | NR | NR | The CARES +UI benefits increased residual savings, reduced recovery time by 6mths (11.8 vs 6.7) and reduced the increase in poverty rates by providing income and benefits to impoverished individuals (CARES: 9.5% -4.5%) | |
| Clay 2021 | USA | survey | food assistance | adult residents in New York | NR | NR | nr | NR | NR | NR | Food assistaance programs were associated with a higher likelihood of experiencing food insecurity | |
| Messer 2021 | USA | survey | incentive for vaccination | adult population in the US | Coupled incentives increase estimated vaccination rates by 7–8%.Offering any incentive yielded an 8% increase (P=0.03) in vaccine uptake compared to the base condition (no incentive), controlling for demographics. Financia Incentive also resulted in a 6% sign decrease in those responding ‘no’ to vaccine uptake and a smaller, nonsignificant decrease in those ‘unsure’. | coupled incentives would bring US vaccination levels from 58% to more than 65% —substantially closer to the rates that experts say are needed to reach herd immunity. | | For Black Americans, 53 per cent (±17%) of respondents in the no-incentive (control) indicated they would choose to receive the vaccine. The $1500 incentive increased vaccine uptake among Black respondents to 68 per cent (±16%). However, for the $2000 incentive, uptake among Black respondents falls dramatically to 39 per cent (±15%). We see a similar trend for Latino respondents, and indeed every racial/ethnic group has a non-linear trend on the size of the incentive. | For income, lower incomes was associated with lower vaccine uptake. The middle-income groups appear most responsive to the incentive. Income quartiles are (1) < $30,000, (2) $30,000 to $59,999, (3) $60,000 to $149,999, and (4) $150,000 or more. | NR | Impact of size of incentive on vaccine uptake: No meaningful difference in the effectiveness of incentives among the three payment levels. | |
| Miyawaki, 2021 | Japan | survey | domestic travel subsidy | domestic travellers in Japan | Participants in the subsidy programme were more likely than non-participants to engage in risky behaviour patterns (visiting restaurants, bars/nightclubs, karaoke bars or fitness clubs at least once) during the state of emergency. As for other preventive measures, participants in the subsidy programme were more likely to use the contact-tracing application and to have received the influenza vaccine in the prior year. See table 2 for impact of program on different public health measures (social distancing, masking, handwashing etc) | After adjusting for potential confounders, we found that participants in the subsidy programme exhibited higher incidence of high fever (adjusted rate, 4.7% for participants vs 3.7% for non-participants; adjusted OR (aOR) 1.83; 95% CI 1.34 to 2.48; p<0.001), sore throat (19.8% vs 11.3%; aOR 2.09; 95% CI 1.37 to 3.19; p=0.002), cough (19.0% vs 11.3%; aOR 1.96; 95% CI 1.26 to 3.01; p=0.008), headache (29.2% vs 25.5%; aOR 1.24; 95% CI 1.08 to 1.44; p=0.006) and smell and taste disorder (2.6% vs 1.8%; aOR 1.98; 95% CI 1.15 to 3.40; p=0.01) compared with non-participants. | NR | The result of the stratified analyses by age showed that the higher incidence rates of COVID-19 like symptoms were more salient among young respondents (online supple- mental table A4).For example, among respondents aged 15–64 years, the adjusted incidence rate of smell and taste disorder was higher for subsidy programme participants compared with younger non-participants, whereas the incidence rates did not differ between participants and non-participants among those aged 65–79 years (p for interaction=0.04). | NR | NR | NR | |
| Raifman, 2020 | USA | survey | unemployment assistance | inidividuals living in households earning less than $75000 in the last 12 months and lost employment at some point during the April to November study period. | NR | NR | NR | NR | NR | NR | Receipt of unemployment insurance was associated with a 4.4 percentage point decline in food insecurity, decline in eating less due to financial constraints. Estimates from event study specifications revealed that reductions in food insecurity and eating less were greatest in the four-week period immediately following receipt of unemployment insurance, with no evidence of differential pre-existing trends in either outcome. | |
| Bhutta, 2020 | USA | survey | mixed | households who benefited from cash assistance by the CARES Act | NR | NR | NR | NR | NR | NR | Availabilty of liquid savings for expenses: We find that the CARES Act dramatically improves households’ financial security. Our estimates suggest that the combination of the Act’s cash assistance and families’ liquid savings would allow nearly all working families (94 percent) to cover their normal recurring expenses if they were to lose all of their income for six months from April through September 2020.2 In contrast, we find that only about half of working families would be able to cover six months of expenses if they had to rely exclusively on their own liquid savings and standard UI benefits. | |
| Men, 2021 | Canada | survey | mixed | non-institutionalized off-reserve popula­tion of the ten provinces of Canada. | NR | NR | NR | NR | NR | NR | Applicants for CERB, regular EI benefits, and other EI benefits had 2.53, 1.80, and 3.01 times higher adjusted odds of food insecurity, respectively, than non-applicants | |
| Fan, 2020 | USA | survey | mixed | working age individuals who were employed and laid off with govt assistance | NR | NR | the currently employed working-age population receiving government assistance has a higher frequency of feeling depressed and hopeless; the currently unemployed working-age population with assistance has a higher frequency of feeling nervous, lonely, and hopeless. | Using the currently employed working-age population without government assistance as a control group (1) both the AA and non-AA currently employed working-age populations with government assistance show no significantly higher or lower frequency of the four symptoms | NR | For the currently employed working-age population with assistance, if residing in non-urban areas, there is no significant effect of having either a higher or lower frequency of mental health conditions compared to the currently employed working-age population without government assistance.However, those who live in urban areas experience a higher frequency of feeling depressed and hopeless. | NR | |
| Madeira, 2021 | Chile | survey | mixed | poor famlies with no formal income, temporarily suspended workers | NR | NR | NR | NR | NR | NR | The income and expenses support were quite significant, representing 13.6% of the average household’s permanent income in April and a higher value of 21.0% in August. Relative to April, the additional measures in August were of great benefit to both the poor and middle class, with benefits increasing from 19.4% to 31.2% of average income for strata 1(strata 1: people with the lowest 50 percentiles of household income as reported in the survey,) and from 10.2% to 18.4% for strata 2 (people between 51-80th percentile of household income). SEE table 1 | |
| Breunig, 2023 | Australia | observational | cash transfer | working‐age population in Australia | NA | NA | NR | NR | NR | NR | 1) the median recipient had 46 per cent of their weekly pre‐COVID‐19 wages replaced by the transfers. 2) a combination of resilience in wage‐earning and the COVID‐19 transfers served to increase total gross income flows among the adult Australian population in 2020. 3) The programs interacted to create a two‐tier welfare safety net that put in place a poverty‐alleviating income floor for workers in low‐earning occupations and those on unemployment benefits, and provided job certainty and greater direct income support to those with higher incomes. | |
| Acharya, 2021 | USA | survey | incentive for vaccination | states residents who receiving vaccine incetive | The augumented synthetic control analysis show that lottery programs were associated with an increase of an average 23.12% increment in the new daily vaccination rate.In state-specific analyses, both methods suggested that the vaccine lottery programs were helpful in Ohio (0.09 log points;P < .001), Maryland (0.26 log points; P < .001), Oregon (0.15 log points; P = .002), and Washington (0.37 log points; P < .001) but not in Arkansas, Kentucky, and West Virginia. The ASC analysis found that lottery programs were positively associated with vaccinations in New Mexico (0.32 log points;P < .001) and New York (0.33 log points; P = .001). | NR | NR | NR | NR | NR | NA | |
| Algara, 2023 | USA | observational | incentive for vaccination | all population in the US | financial incentives positively influence vaccine preferences among the mass public and all partisan groups, including Republicans initially “unlikely” to be vaccinated. Using the observational data, we replicate our experimental findings showing positive financial incentive attitudes positively correlate with self-reported vaccination disclosures. | NR | NR | NR | NR | NR | NR | |
| Andersen, 2022 | USA | survey | expanded paid sick leave | working class population | We find that the policy leads to increased self-quarantining as proxied by staying home.FFCRA leads to a 0.38 h or 22.9-min decrease in average time away from home and a 1.8 ppt decrease in the share of devices away from home eight more hours per day. | We also find that COVID-19 confirmed cases decline post-policy.We can also put this decline in context by comparing our estimated change in cases to the change in mobility. Our findings suggest that the weekly COVID-19 incidence decreased by 7.7 log points post-FFCRA, using a one standard deviation change in the nonessential establishment share. | NR | NR | NR | NR | NR | |
| Barr, 2021 | USA | survey | food assistance | individuals who utilized free meal services in Kentucky during the COVID-19 pandemic. | NR | NR | changed habits (e.g., healthier eating; n = 17), (2) mental wellbeing (e.g., reduced stress regarding food intake; n = 27), | NR | NR | NR | provided resources (e.g.,extra food, budgeting; n = 59), and | |
| Batra, 2023 | USA | quasi experimental | expanded child tax credit | low-and middle income families, unemployed parents | NR | NR | fewer depressive and anxiety symptoms among low-income adults.(13.3 percent reduction from baseline anxiety levels (25.5 percent) | Adults of Black, Hispanic, and other racial and ethnic backgrounds demonstrated greater reductions in anxiety symptoms compared to non-Hispanic White adults with children. | NR | NR | NR | |
| Choi, 2022 | Korea | survey | cash transfer | Gyeonggi provincial government residents | NA | NA | NA | NA | NA | NA | the stimulus payments led to significant increases in card spending in establishments accepting the Gyeonggi local currency (ie increased local consumption), relative to other establishments. The estimated overall spending effect of 4.1% persisted over three weeks, and the effects are heterogeneous across sectors. | |
| Jacobson, 2022 | USA | randomized controlled trial | incentive for vaccination | vaccine hesitant population | financial incentives and the health consequences videos rule out increases in vaccination rates larger than 1.0 and 1.6 percentage points, respectively. | NR | NR | financial incentives reduced vaccination rates in both older individuals (ages 40 and over) and those who indicated they supported Trump in the 2020 presidential election.( For respondents ages 40 and over, 30-day vaccination rates declined by 4.5 percentage points (p = 0.045) and 4.7 percentage points (p = 0.041) in response to the $10 and $50 incentives, respectively. For respondents who indicated they supported Trump, the $50 incentive decreased vaccination rates by 4.2 percentage points (p = 0.047)) | NR | NR | NR | |
| Jun'd, 2022 | Australia | survey | incentive for vaccination | all population | Those who entered the competition were 2.27 (95% CI 1.73 to 2.99) times more likely to be vaccinated after the competition opened on 1 October than those who did not enter—an increase in the probability of having any dose of 0.16 (95 % CI 0.10 to 0.21) percentage points. This increase was mostly driven by those receiving second doses. Entrants were 2.39 (95% CI 1.80 to 3.17) times more likely to receive their second dose after the competition opened. | NR | NR | NR | NR | NR | NR | |
| Kim 2021 | USA | survey | food assistance | working-aged Americans with job-related income loss during recent months of the pandemic and housing renters. | NR | NR | Experiencing considerable financial hardship (vs no hardship) predicted nearly 3-fold higher risks of anxiety and depressive symptoms (e.g., adjusted prevalence ratio, PR of depression = 2.75, 95% CI = 2.54–2.98, P < .001), | NR | NR | NR | Financial hardship rose progressively from September to December 2020, and disproportionately affected Black non-Hispanic and Hispanic Americans and lower-income households. a 23-fold higher risk of food insufficiency (PR = 22.71, 95% CI = 15.62–33.01, P < .001), and a 27-fold higher risk of a likely eviction (PR = 27.20, 95% CI = 10.63–69.59, P < .001). working-aged adults experiencing financial hardship had markedly greater risks of anxiety or depressive symptoms, food insufficiency, and an anticipated housing eviction. | |
| Li, 2022 | USA | survey | food assistance | all population | NA | NA | NA | NA | NA | NA | SNAP significantly lowered the odds of being food insufficient by 24.5% among households who were already food insufficient before the pandemic and by 11.9% for households with children. FAPs for children slightly reduced FI among households with children, but those who participated in these programs still had higher prevalence of food insufficiency than those who did not. Community FAPs were not effective. | |
| Liu, 2023 | USA | survey | cash transfer | pre-retiree US residents who were younger than 65 and were interviewed in the November 2020 COVID-19 supplement of the HRS. | Negative association between the amount of stimulus received and financial hardship experienced by respondents during the pandemic. Financial wealth was negatively associated with the perception of feeling depressed. | NR | Negative association between the amount of stimulus received and financial hardship experienced by respondents during the pandemic. Financial wealth was negatively associated with the perception of feeling depressed. | African American households were less likely to increase spending, Hispanic households were more likely to increase savings. | households with lower educational attainment were more likely to pay down debt using their stimulus money. | NA | NA | |
| Lowery, 2022 | USA | survey | food assistance | households participating in the Supplemental Nutrition Assistance Program (SNAP) at the chain between October 2019 and December 2020.We | NA | NA | NA | NA | NA | NA | Healthy Helping enrollment was associated with a $26.95 increase in monthly spending on fruit, vegetables, nuts, and legumes—an increase of 2.5 grams of fiber per 1,000 kilocalories purchased—and other shifts in the composition of food purchases, relative to control shoppers. These findings suggest that the program increased healthy food purchases while also increasing dollar sales at participating retailers. | |
| Park, 2022 | USA | survey | unemployment assistance | jobless mortgage borrowers who received or waited for UI benefits between August 2020 and May 2022. | NA | NA | NA | NA | NA | NA | Minority borrowers were more likely to have a difculty in paying mortgage than White borrowers. UI recipients—regardless of race and ethnicity—were less likely to experience mortgage difculties, but the positive unemployment beneft was reduced disproportionately among Blacks. Blacks were also at a higher risk of mortgage difculties compounded by other pandemic-induced hardships—loss of household, lack of food, and mental illness—even after the receipt of UI. | |
| Pilkauskas, 2023 | USA | randomized controlled trial | cash transfer | families with low income receiving, or had recently received, Supplemental Nutrition Assistance Program benefits. | NA | NA | no statistically significant effects of the cash transfer and mental health | NR | NR | NA | NA | |
| Pollack, 2022 | USA | observational | expanded paid sick leave | all population | States with pre-existing PSL policies exhibited a greater drop in mobility following the passage of the FFCRA (b 1⁄4 8.86, 95% confidence interval: 11.6, 6.10, P < 001). This remained significant after adjusting for state-level health, economic, and sociodemographic indicators (b 1⁄4 3.13, 95% confidence interval: 5.92, 0.34; P 1⁄4 .039). | NR | NR | NR | NR | NR | NR | |
| Shmueli, 2022 | Isreal | survey | incentive for vaccination | all adult population sample | Incentives such as monetary rewards or the green pass did not increase the probability of getting vaccination immediately. | NR | NR | NR | NR | NR | NR | |
| Wahdat, 2022 | USA | survey | cash transfer | late recipients of Economic impact papyment | NR | NR | NR | NR | NR | NR | EIPs were associated with a 9.2 percentage points decrease in the likelihood of food insufficiency. | |
| Dudley, 2021 | USA | survey | incentive for vaccination | pregnant women receiving prenatal intervention | Self- reported receipt of influenza vaccine-- The provision of small pharmacy-based financial incentives combined with individually- tailored educational videos about vaccines led to 6.97 (95%CI: 2.25–21.64) times higher odds of self- reported receipt of influenza vaccine than providing small pharmacy-based financial incentives without these videos. Provision of small pharmacy-based financial incentives ONLY had 0.22 (95%CI: 0.09– 0.51) times the odds of reporting receiving influenza vaccine from any location compared to the control group( provision of no educational video & financial incentive). | NR | NR | NR | NR | NR | NR | |
| Yue, 2021 | Singapore | randomized controlled trial | incentive for vaccination | senior adult population(65 years of age or older ) | VACCINATION-- Increasing the total incentive for vaccination and reporting from 10 to 20 SGD increased participation in vaccination from 4.5% to 7.5% (P , .001). Increasing the total incentive from 20 to 30 SGD increased the participation rate to 9.2%, but this was not statistically significantly different from a 20-SGD incentive | NR | NR | The group of nonworking elderly were more sensitive to changes in incentives than those who worked(P=<0.001). There were no significant differential effects by age group, gender, however. | The effects of increasing incentives on influenza vaccination rates differed by ethnicity (P=0.001), socio-economic status(P=<0.0001), household size (0.009), and a measure of social resilience (<0.001). here were no significant differential effects by education, however | NR | NR | |
| Zhai, 2018 | USA | survey | expanded paid sick leave | Adults aged 18 years and older who were employed full time | Vaccination: workers with paid sick leave (PSL) were more likely to be vaccinated, (with influenza vaccination coverage estimates for workers with PSL approximately 10-15% higher) than those without PSL, respectively. (fig1) | NR | Seek care from health professionals: PSL beneficiaries were more likely to seek treatment for their illness than those who did not have PSL (48.9% vs. 30.7%). Multivariable analysis confirmed that having  PSL benefits was independently associated with seeking treatment for their illness Adjusted prevalence ratio 1.21 (1.01, 1.44) (Table 2). | NR | NR | NR | NR | |
| Asfaw, 2018 | USA | survey | expanded paid sick leave | families and individuals employed in private sector and its employees. | Potential Physical distancing: workers with PSL spent an average of 4.58 days away from work per year due to illness or injury, while workers without PSL spent 3.48 [or 1.10 fewer (95% CI: 0.90 to 1.30)] days away from work, controlling for all covariates in the model | Assuming 10% to 12% of days absent due to ILI, 0.026% transmission rate (adjusted for flu vaccination and vaccination effectiveness rate), and an average daily contact of three to five coworkers, a worker at work due to lack of PSL could infect on the average 0.0405 to 0.0810 coworkers per year. | NR | NR | NR | NR | Providing PSL could have saved employers $0.63 to $1.88 billion in reduced ILI- related absenteeism costs per year during 2007 to 2014 in 2016 dollars. | |
| Wilson, 2014 | USA | survey | expanded paid sick leave | working adults aged 18 and above | VACCINATION-The proportion of workers receiving vaccination annually was higher for those with paid leave versus without paid leave (34.0% vs. 21.0%, P < 0.001). Adjusted odds of having a vaccination increased with paid leave vs. without paid leave (OR = 1.42, CI: 1.31–1.53) | Paid leave decreased in number of flu cases by 57 thousand | Universal paid leave is predicted to result in 18.2 thousand fewer healthcare visits, for the flu annually. | White non-Hispanics had higher odds of flu vaccination from paid leave than another race/ethnicity (OR: 1.43 vs. 1.38, respectively) | Paid leave had a larger impact on respondents who were not in poverty compared to in-poverty (OR: 1.44 vs. 1.23, respectively) and who had excellent/good versus poor health status (OR: 1.43 vs.1.38, respectively). However, differences were not statistically significant across these groups | NR | Universal paid leave was predicted to avert 63.8 thousand workdays lost to influenza each year, accounting for 10.3 million dollars in wage income. | |

*Legend: NR = not reported, NA =not applicable*
